# Applications of Chitosan in Prevention and Treatment Strategies of Infectious Diseases

**DOI:** 10.3390/pharmaceutics16091201

**Published:** 2024-09-13

**Authors:** Genada Sinani, Melike Sessevmez, Sevda Şenel

**Affiliations:** 1 Department of Pharmaceutical Technology, Faculty of Pharmacy, Altinbas University, 34147 Istanbul, Türkiye; genada.sinani@altinbas.edu.tr; 2 Department of Pharmaceutical Technology, Faculty of Pharmacy, Istanbul University, 34116 Istanbul, Türkiye; melike.sessevmez@istanbul.edu.tr; 3 Department of Pharmaceutical Technology, Faculty of Pharmacy, Hacettepe Univesity, 06100 Ankara, Türkiye

**Keywords:** chitosan, vaccine, infections, delivery system, nanoparticles

## Abstract

Chitosan is the most commonly investigated functional cationic biopolymer in a wide range of medical applications due to its promising properties such as biocompatibility, biodegradability, and bioadhesivity, as well as its numerous bioactive properties. Within the last three decades, chitosan and its derivatives have been investigated as biomaterials for drug and vaccine delivery systems, besides for their bioactive properties. Due to the functional groups in its structure, it is possible to tailor the delivery systems with desired properties. There has been a great interest in the application of chitosan-based systems also for the prevention and treatment of infectious diseases, specifically due to their antimicrobial, antiviral, and immunostimulatory effects. In this review, recent applications of chitosan in the prevention and treatment of infectious diseases are reviewed, and possibilities and limitations with regards to technical and regulatory aspects are discussed. Finally, the future perspectives on utilization of chitosan as a biomaterial are discussed.

## 1. Introduction

**Figure 1:**
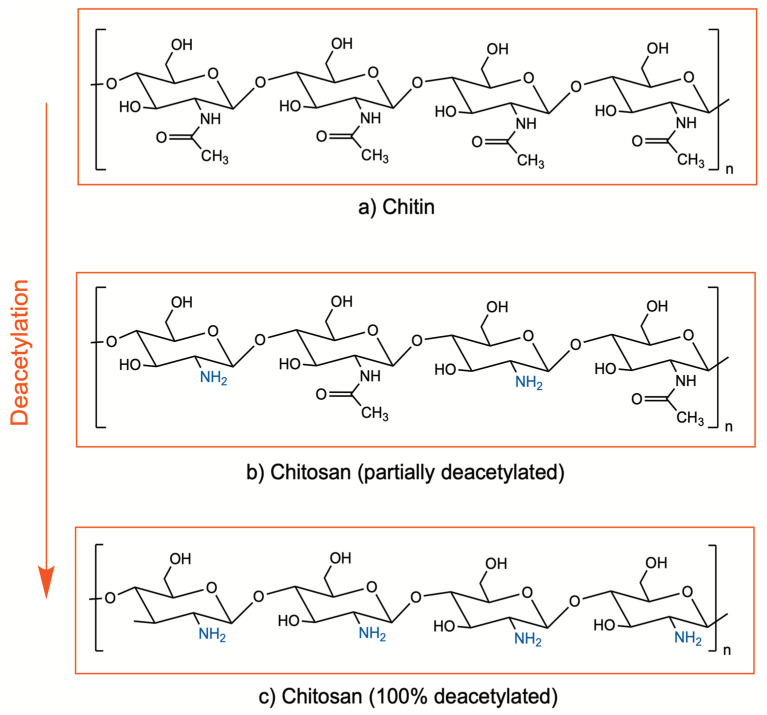
Chemical structure of chitin (**a**) and chitosan (**b**,**c**).

Chitosan is a cationic biopolymer being investigated in a wide range of medical applications due to its promising properties such as biocompatibility, biodegradability, bioadhesivity, and bioactive properties. It is composed of β(1–4)-linked d-glucosamine and N-acetyl-*d*-glucosamine units and obtained by deacetylation of chitin, which is found in the exoskeletons of crustaceans, mollusks, and algae, as well as insects and the cell walls of fungi [1,2]. Enzymatic or chemical processes at alkali conditions are applied for deacetylation of chitin, in order to remove the acetyl groups from the molecular chain of chitin, resulting in free amino groups (–NH_2_). Currently, the chemical process is preferred due to cost-effectiveness and suitability for large-scale production. The degree of deacetylation indicates the ratio between the D-glucosamine (deacetylated) and N-acetyl-*D*-glucosamine (acetylated) units of chitosan (Figure 1). Although there is no precise definition for the deacetylation degree, chitin with a deacetylation degree of approximately 65–70% and higher is generally referred to as chitosan [2,3,4].

The bioactive properties of chitosan include mainly antimicrobial, antiviral, anti-inflammatory, hemostatic, and immunostimulatory [2,5,6,7,8,9]. Furthermore, it has been shown to enhance the epithelial permeability [10]. Different mechanisms have been suggested to explain the permeation enhancement of chitosan. In early years, the permeation enhancement was attributed to its bioadhesive property, which prolongs the attachment of the drug at the application site as well as to its effect on the lipid organization of the cell membrane [11,12]. Recent studies showed that penetration enhancement was through tight junction disruption at gene and protein expression levels [13]. Furthermore, interaction between the quaternized amine groups of chitosan and cell membranes plays a crucial role in its biological activities.

**Figure 2:**
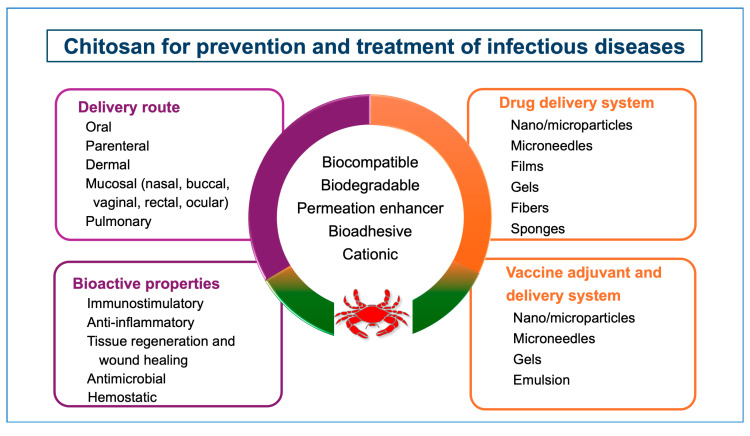
Potential of chitosan for prevention and treatment of infectious diseases.

Chitosan has been investigated as a drug delivery system for the treatment of numerous diseases, including infectious diseases as well. Its versatile physicochemical (surface charge, molecular weight, etc.) and biological (mucoadhesion, penetration enhancement) properties allow the formulators to develop customized drug delivery systems in different forms such as hydrogels, nanofibers, micro-and nanoparticles, films, fibers, etc. (Figure 2) [2,14,15,16,17,18,19,20,21]. Chitosan-based delivery systems can be administered via different routes, such as oral, parenteral, mucosal, dermal, and pulmonary, providing efficacy and safety [22,23,24].

In the following sections, after summarizing the properties of chitosan that have an impact on a successful drug and vaccine delivery system for prevention and treatment of the diseases, applications of chitosan-based systems for the treatment and prevention of various infectious diseases, including SARS-CoV-2, will be reviewed in regard to both its own bioactive properties and for delivery of drugs and antigens.

## 2. Chitosan and Drug Delivery

The source, degree of deacetylation, and molecular weight (Mw) of chitosan are considered to be important parameters since they affect the physicochemical and biological properties of chitosan, including its solubility, biocompatibility, biodegradation, bioadhesion, and biological activities [25,26,27,28,29,30]. The presence of protonated amino groups in chitosan determines its solubility in weak acid solutions with a pKa value of approximately 6.5. The solubility of chitosan depends on its degree of deacetylation as well as the pH of the medium. With the higher degree of deacetylation, chitosan becomes more soluble, as a high degree of deacetylation leads to an increased number of free amino groups in the molecular chain of chitosan [31]. Chitosan can be obtained in a wide range of molecular weights, generally classified as high molecular weight chitosan (>1000 kDa), medium molecular weight chitosan (150–1000 kDa), and low-molecular-weight chitosan (<100 kDa). Chitosan with a higher molecular weight shows less solubility due to increased interactions among the hydrogen bonds. Chitosan oligosaccharides (COS) are degraded by depolymerization from chitosan using a chemical or enzymatic hydrolysis procedure [32].

Due to their higher solubility and enhanced bioactive properties, COS have been widely investigated in biomedical applications [33,34]. Water-soluble chitosan was obtained by forming its salt form with glutamic, aspartic, lactic, and glycolic acids [35]. Solubility of chitosan can be increased also by producing its derivatives by modifications through its functional groups (free amino and hydroxyl groups) via chemical reactions such as acylation, carboxylation, alkylation, sulfonation, quaternization, etc. [36,37,38]. The physicochemical and bioactive properties of the chitosan remain the same with these derivatives, while exerting new and/or improved characteristics depending on the nature of additional functions [39,40,41,42,43,44,45,46,47].

Due to its cationic nature and penetration-enhancing and bioadhesion properties, chitosan as a delivery system provides enhancement of the permeability of the tissue to the drug, increased contact time of the drug at the application site, targeted delivery of the drug to the desired area, and controlled/prolonged drug release. It is also possible to reduce undesirable side effects during treatment with tailored delivery systems, especially with nanoparticulate systems. Aside from the advantages mentioned above, a more important property of the chitosan-based delivery systems in the treatment or prevention of infectious diseases is the antiviral, anti-inflammatory, and antimicrobial activity of chitosan itself.

Chitosan is commercially available from different suppliers worldwide in various molecular weights, degrees of deacetylation, viscosity, and purity grades. However, for drug and vaccine delivery, the quality and safety of the chitosan are crucial requirements. Currently, pharmaceutical-grade chitosan is described in various pharmacopeia, including Japanese, European, United States, etc., which is an animal source (specified as shells of shrimp and crabs), with a degree of deacetylation required to be above 70% [48,49]. The other specifications of chitosan stated in the pharmacopeia are appearance, solubility, identification (infrared, reaction of chlorides, gelatinous mass formation), bacterial endotoxin, microbial limits, loss on drying, residue on ignition (or sulphated ash), limit of heavy metals (lead, mercury, chromium, nickel, cadmium, arsenic), limit of protein content, limit of iron, average molecular weight and molecular weight distribution, appearance of solution, matter insoluble in water, pH, viscosity. Most of these parameters have been shown to have an impact on the efficacy and safety of chitosan and chitosan-based delivery systems. It is important that chitosan meets the compendial requirements to ensure the safety and efficacy of chitosan-based systems. For example, being a naturally derived polymer, it may contain bacterial endotoxins (lipopolysaccharides), which can cause undesired inflammatory responses [50].

Currently, there is no approved drug delivery system that contains chitosan or its derivatives. The products containing chitosan that are on the market are medical devices that are used only externally, mainly as hemostatic dressings in different forms. Nevertheless, numerous studies are being performed, with few reaching to the clinical trials for drug and vaccine delivery for protection and treatment of infectious diseases [51,52]. The recent clinical trials based on chitosan related to treatment and prevention of infections that are listed in ClinicalTrials.gov are summarized in Table 1.

## 3. Recent Applications of Chitosan in Drug Delivery against Infectious Diseases

Infectious diseases caused by pathogenic microorganisms, such as bacteria, viruses, parasites, or fungi, continue to be the major causes of illness, disability, and death. New infectious diseases are being identified over time, and some diseases that were previously considered under control are reemerging as well [53]. Furthermore, antimicrobial resistance (AMR) became among the top ten global public health challenges [54]. Hence, humanity still faces big challenges in the prevention and control of infectious diseases, which is crucial to ensuring healthy individuals and healthy societies. By means of designing suitable delivery systems, it is possible to improve the efficacy of the current antimicrobial treatments and provide an option to avoid or mitigate the antimicrobial resistance as well. In this regard, chitosan exerts great potential for its bioactive properties, especially its antimicrobial activity against gram-positive and gram-negative bacteria, viruses, parasites, and fungi [5,55,56,57,58]. It has been widely investigated as an antimicrobial agent for treatment of various infectious diseases [5,59,60].

Disruption of the bacterial cell membrane, interaction with intracellular targets, chelating of metal cations, and alteration of the cell permeability of microbes are among the proposed antibacterial mechanisms of chitosan [61,62]. Antimicrobial activity of chitosan has been shown to depend on its physicochemical characteristics (Mw and DD), type of microorganism, origin of chitosan, pH, concentration, and experiment conditions [63,64,65]. A consensus has not yet been reached on the relationship between antimicrobial activity and the properties of chitosan. The relation between the antimicrobial activity and the molecular weight of chitosan has been shown to be influenced by the type of implied pathogen [27]. In our previous study, in which chitosan with different molecular weights (150–5000 kDa) were compared, the high molecular weight chitosan (500–5000 kDa) was found to be the most effective against *Porphyromonas gingivalis*, *Aggregatibacter actinomycetemcomitans*, and *Candida albicans* strains [17]. On the other side, as the pH of the high molecular weight chitosan used in this study was lower (pH 3.5) than that of the low (pH 5.5) and medium (pH 4.0), molecular weight chitosans, our results also demonstrated that the antimicrobial activity of chitosan is inversely affected by pH, with higher activity observed at lower pH values, with the amino groups of chitosan protonated and converted to the quaternary form. On the other hand, water-soluble chitosan derivatives, which have a pH higher than that of chitosan, have been shown to exert enhanced antimicrobial activity [66,67,68,69]. Furthermore, it was reported that depending on the antimicrobial activity assay (e.g., agar dilution, broth dilution) performed, the results on the antimicrobial activity of chitosan would show differences [70].

The antibacterial activity of chitosan against Gram-positive *Staphylococcus aureus (S. aureus)*, which causes commonly skin infections, and Gram-negative *Escherichia coli (E. coli)*, which frequently infects the gastrointestinal and urinary tract, has been demonstrated in a significant number of studies. Chitosan in nanoparticulate form at 10 and 20% concentration could completely inhibit the growth of *Streptococcus mutans (S. mutans)*, *Pseudomonas aeruginosa* (*P. aeruginosa*), and *Enterococcus faecalis (E. faecalis)* in 24 h and 48 h [71]. In another study, nanoparticles prepared at neutral pH using chitosan with a molecular size lower than kDa and a deacetylation degree lower than 60% showed increased antibacterial activity against *S. mutans* biofilms, and the antibacterial effect decreased when high molecular weight chitosan was used [72].

Chitosan derivatives such as carboxymethyl chitosan [73], *N*,*N*,*N*-trimethyl chitosan [74], 2′-*O*-hydroxypropyl trimethyl ammonium chloride chitosan [75], and sulfonated chitosan [76] have been reported to exert enhanced antibacterial activity compared to unmodified chitosan against different pathogenic bacteria and strains, e.g., *E. coli*, *Klebsiella* spp., *P. aeruginosa*, and *Bacillus subtilis (B. subtilis)*. Combination of chitosan with materials that exert antibacterial activity is also widely used to enhance its antibacterial activity [77,78,79]. It was shown that when chitosan in nanoparticle form was prepared using cinnamaldehyde as a crosslinker instead of the commonly used crosslinker, tripolyphosphate (TPP), the antibacterial activity against *S. aureus* and *E. coli* was enhanced significantly [77].

Apart from its antimicrobial activity, chitosan has been investigated as a delivery system of drugs in different forms, such as micro- and nanoparticles, hydrogel, aerogel, patch, fiber, sponge, etc., for the treatment of infectious diseases [19,56,80,81,82,83]. Recent applications of chitosan delivery systems in the treatment of infectious diseases are summarized in the following sections.

### 3.1. Bacterial Infections

Bacterial infections have a large impact on public health. Bacteria can be transmitted to humans through air, water, food, or living vectors. The disease can occur at any part of the body after being infected by the microorganism. The rise of antimicrobial resistance and a decreasing new antimicrobial pipeline have been recognized as emerging threats to public health. The ESKAPE pathogens (*Enterococcus faecium*, *S. aureus*, *Klebsiella pneumoniae*, *Acinetobacter baumannii*, *P. aeruginosa*, and *Enterobacter* spp.) continue to be critical multidrug-resistant bacteria for which effective therapies are rapidly needed, despite the introduction of several new antibiotics and alternative molecules or approaches to antibiotic treatment [84,85].

Furthermore, chitosan and its derivatives have been successfully employed for the delivery of antimicrobial agents, including polyphenolic compounds such as tannic acid [86], plant essential oils or plant extracts [87,88], as well as several antibiotics such as rifampicin, ciprofloxacin, vancomycin, and doxycycline [89].

In addition to its antibacterial effect and suitable properties as a drug delivery system, chitosan-based delivery systems can provide controlled release of the drug, which further improves the efficacy of the treatment of bacterial infections. Key findings from recent studies utilizing chitosan for delivery of different antibacterial agents against pulmonary, dental, vaginal, etc. infections are summarized in Table 2.

**Table 2:** Summary of chitosan-based delivery systems against bacterial infections reported in recent years.

Drug	Chitosan Properties	Delivery System	Aim	In Vitro Studies	In Vivo Studies	Results	Ref.
Chlorhexidine digluconate	Chitosan (Mw: 550 kDa, DD: 75%)	Composite silk fibroin/chitosan hydrogel	Wound dressing	–Antibacterial activity against *E. coli* and *S. aureus* –Cytotoxicity in HSF cells –Coagulation assay –Drug release		–Enhanced antibacterial activity –80% relative cell proliferation –Shorter blood clotting time –Enhanced coagulant effect with increased chitosan amount –50% release for 24 h	[90]
Amoxicillin, cefuroxime, tetracycline	Chitosan (DD: 78%)	Chitosan-PEG hydrogel	Wound dressing	–Antibacterial activity against *S. aureus* and *E. coli* –Anti-inflammatory activity in THP-1 macrophages –Toxicity in MRC-5 human fibroblast cells –Ex vivo wound-healing study in ulceration model using hOSECs	Acute systemic toxicity testing in mice	–High antibacterial activity –Reduced levels of pro-inflammatory TNF-α –Biocompatible –No systemic toxicity –Higher wound healing	[91]
Silver	Hydroxyethylacryl chitosan synthesized from chitosan extracted from shrimp shells with MW: 330 kDa, DD: 85%	Hydroxyethylacryl chitosan/ sodium alginate film	Wound dressing	–Antibacterial activity against *S. aureus* and *E. coli* –Cytotoxicity (MTT) in Vero cells		–Biocompatible –High antibacterial activity	[92]
Tannic acid	Chitosan (DD: 95%)	Chitosan/sulfated polysaccharides bilayer on poly(l-lactic acid) film	Antibacterial	–Antibacterial activity against *E. coli* and *S. aureus* –Hemocompatibility and blood clotting time –Cytotoxicity in L929 cells		–Enhanced antimicrobial activity –Increased hemocompatibility –Blood clotting in 21s –Biocompatible	[93]
Minocycline Rifampicin	Chitosan (DD: 85%)	Chitosan-ECM hybrid scaffolds	Wound dressing	–Antibacterial activity against *E. coli* and *S. aureus* –Cytotoxicity (neutral red uptake) in HMEC-1		–Enhanced antimicrobial activity –Lower direct cytotoxicity with chitosan-containing scaffolds compared to ECM alone	[94]
Vancomycin hydrochloride	Chitosan (Mw: 200–400 kDa, DD: 90%)	Chitosan aerogel beads (∼1.5–4.5 mm)	Chronic wound healing	–Antimicrobial activity against *S. aureus* –Collagenase activity –Water sorption capability –Drug release –Cytocompatibility in BALB/3T3 fibroblasts		–Enhanced antibacterial activity –No inhibition of collagenase activity, indicating no interaction with the normal biological healing process –High water absorption capacity –Biocompatible –Initial fast release followed by slower release rate	[95]
Rifampicin Ciprofloxacin Vancomycin Doxycycline	Chitosan (Mw: medium, DD: 85%)	Chitosan-propolis nanoparticles (247 nm)	Biofilm inhibition	–Antibacterial activity against *S. epidermidis* –Biofilm-related gene expression analysis		–Disruption of biofilm and significant decrease in bacterial viability –Downregulation of biofilm-related genes	[96]
Bacteriophage cocktail of *S. enterica*, *Sh. flexneri*, and *E. coli*	Chitosan	Chitosan nanoparticles (298 ± 2 nm)	Bacterial diarrhea	–Drug release in pH 1.2 and pH 6.8	Model: Gastrointestinal infection model in rats Dose: 10^10^ PFU/mL of phage cocktail orally gavaged daily	–Protection of phage in simulated gastric acidic fluid –No weight loss in 3 days –Growth inhibition	[97]
Clarithromycin	Chitosan	Chitosan nanoparticles (152 ± 5 nm)	Ocular infection	–Antibacterial activity against *S.aureus* and *P. aeruginosa* –Ex-vivo permeation study using goat cornea –HET-CAM test to evaluate ocular irritation –Bioadhesion –Drug release in simulated tear fluid		–Enhanced antibacterial activity –2.7-fold higher corneal permeation –No damage on excised goat cornea –Mucoadhesion –Sustained drug release	[98]

DD: deacetylation degree; *E. coli: Escherichia coli*; ECM: extracellular matrix; HET-CAM: hen’s egg chorioallantoic membrane; HMEC-1: Human microvascular endothelial cells; kDa: kilodalton; mL: milliliter; Mw: molecular weight; nm: nanometer; *P. aeruginosa: Pseudomonas aeruginosa*; PEG: poly(ethylene glycol); PFU: plaque-forming units; *Staphylococcus aureus: S. aureus; S. enterica: Salmonella enterica; S. epidermidis: Staphylococcus epidermidis; S. flexneri: Shigella flexneri*.

### 3.2. Fungal Infections

Fungal pathogens are still a major threat to public health as they are becoming increasingly common and resistant to treatment with limited antifungal medicines currently available [99]. In 2022, WHO published a report highlighting the first-ever list of fungal “priority pathogens” consisting of 19 fungi (e.g., *Candida albicans* and other *Candida* spp., *Aspergillus fumigatus*, *Fusarium* spp.) that represent the greatest threat to public health [100]. In this report, besides the fungal priority pathogens list (FPPL), the unmet research and development needed to strengthen the global response to fungal infections and antifungal resistance was also emphasized.

*Candida albicans (C. albicans)* and other *Candida* spp., *Aspergillus fumigatus (A. fumigatus)*, *and Fusarium* spp. Are among the 19 fungal pathogens requiring prioritized research, development, and public-health actions [100]. The antifungal activity of chitosan-based delivery systems has been demonstrated against *Candida* spp., including *C. albicans* [101,102,103], *Aspergillus niger* (*A. niger*) [104,105,106], *A. fumigatus* [107], and other fungi such as *Cryptococcus neoformans*, *Kodamaea ohmeri*, etc. [55,108]. Although the antifungal mechanism of chitosan is not completely clear, leakage of cellular components because of interaction with the fungal cell membrane, binding of essential cell nutrients, and interaction with genetic material have been proposed as potential mechanisms [109,110,111].

Similar to the factors influencing the antibacterial activity of chitosan, its antifungal activity is also affected by the physicochemical properties of chitosan (e.g., molecular weight and deacetylation degree), concentration, and fungi type [106,109,112,113]. Additionally, antifungal effects have been shown to be improved with chitosan derivates such as carboxymethyl chitosan [114], sulfanilamide derivatives [115], and N-(2-Hydroxypropyl)-3-trimethylammonium chitosan chlorides [55]. Some fungi are inherently chitosan resistant, as it is naturally found in their cell walls [113]. Particularly, *A. niger* was shown to be more chitosan resistant compared to *Fusarium solani* and *C. albicans* [106], and alternative approaches may be required to overcome resistance. For example, encapsulation of clove essential oil as an antifungal agent in chitosan nanoparticles improved the antifungal effect against *A.niger* compared to unencapsulated clove oil or chitosan nanoparticles alone [116]. As a result of its good antifungal properties, in addition to applications in the pharmaceutical field, chitosan is widely evaluated as an antifungal agent in the food, cosmetic, and agricultural industries. Recent studies focusing on the use of chitosan for delivering antifungal agents against human fungal pathogens are summarized in Table 3.

**Table 3:** Summary of chitosan-based delivery systems against fungal infections reported in recent years.

Drug	Chitosan Properties	Delivery System	Aim	In Vitro Studies	In Vivo Studies	Results	**Ref.**
Clotrimazole	Chitosan (Mw: 1087 kDa, DD: 92%)	Powder-solid mixtures of drug-chitosan	Antifungal	Antifungal activity against *C. glabrata*		–Enhanced antifungal effect	[117]
Clotrimazole	Chitosan (low Mw: 50–190 kDa, medium Mw: 190–310 kDa, high Mw: 310–375 kDa)	Wafers	Vaginal candidiasis	Drug release	Model: Vaginitis model by *C. albicans* suspension (1 × 10^6^ CFU/mL) –Antifungal activity	–Sustained drug release for 8 h –Enhanced *C. albicans* inhibition (89%) with low Mw chitosan wafers compared to marketed product (76.30%).	[118]
Metronidazole	Chitosan (ChitoClear^®^) (Mw: <50 kDa, DD: 90%)	Chitosan dispersion Chitosan nanoparticles in HPMC hydrogel	Vaginal -infections	Antifungal activity against Candida spp. (*C. albicans* and non-*albicans Candida* strains)		–Enhanced antifungal activity in the presence of chitosan –Antimicrobial activity dependent on *Candida* species	[119]
Oxiconazole	Chitosan	Chitosan/carboxymethylcellulose/scleroglucan/montmorillonite thermosensitive nanocomposite hydrogel	Onychomycosis	–Antifungal activity against *T. mentagrophytes* and *T. rubrum* dermatophytes –Drug release		–High antifungal activity –>50% drug release for 7 h, depending on montmorillonite concentration	[120]
Nystatin	Chitosan glutamate	Nystatin in chitosan solution	Oral candiasis		Model: Oral candidiasis model in rats induced by inoculum *C. albicans* at 3 × 10^8^ CFU/mL on the dorsal tongue –Treatment dose: 100,000 IU/mL nystatin –Evaluation of lesions	–Combination of nystatin with chitosan improved therapeutic response compared to commercially available orabase at both 5 and 10 days after infection	[121]
Nystatin	Chitosan	Chitosan-coated iron oxide nanoparticles as (10–20 nm)	Local fungal infections	–Antifungal activity against *Candida* spp. and *A. fumigatus* –Cytotoxicity on the adipose-derived mesenchymal stem cells		–Enhanced antifungal effect –No toxicity	[122]
Miconazole	Chitosan	Chitosan nanoparticles (245 nm)	Vulvovaginal candidiasis		Model: Vulvovaginal candidiasis murine model with *C. albicans* –Fungal burden assay –Cytokine assay –Biochemical and genotoxicity analyses	–No toxicity –Reduced TNF-α and IL-10 levels –Higher antifungal activity compared to commercial product, which contains higher concentration of drug	[123]
Rose Bengal	Chitosan (Low Mw: 50–190 kDa; DD: 75%)	Chitosan nanoparticles (200 nm)	Onychomycosis	–Antifungal activity against *T. rubrum*, *T. mentagrophytes*, and *T. interdigitale* –Biocompatibility on MRC-5 cells –Uptake by *T. rubrum* spores		–Enhanced fungal inhibition –No toxicity –Enhanced uptake	[124]
Ciprofloxacin and fluconazole	Chitosan (Mw: 100–150 kDa; DD: 85%)	Fibrin-nanoparticle-incorporated chitosan bandages (hydrogel)	Wound dressing against polymicrobial infections	–Antimicrobial activity against *C. albicans*, *E. coli*, *and S. aureus* –Cytocompatibility in HDF –Drug release	Model: Infected rat wound model by 10^6^ CFU of *S. aureus*, 10^6^ CFU of *E. coli*, and 10^7^ CFU *C. albicans*	–Enhanced antimicrobial activity –Significant reduction in microbial load in vivo –Biocompatible –Sustained drug release for 14 days	[125]

*C. albicans: Candida albicans*; *C. glabrata: Candida glabrata*; *A. fumigatus: Aspergillus fumigatus*; CFU: colony-forming unit; DD: deacetylation degree; *E. coli: Escherichia coli*; kDa: kilodalton; mL: milliliter; Mw: molecular weight; nm: nanometer; *S. aureus: Staphylococcus aureus*; *T. mentagrophytes: Trichophyton mentagrophytes*; *T. rubrum: Trichophyton rubrum*; *T. interdigitale: Trichophyton interdigitale*.

### 3.3. Viral Infections

The infectious viruses invade living normal cells and use these cells to reproduce themselves. They cause mild to severe infections in humans, which some may lead to death if not treated [126]. Viral infections involve the nose, throat, and upper airways, or systems such as the nervous, gastrointestinal, and reproductive systems. Amongst the common virus diseases are cold and flu, but in recent years, COVID-19, Ebola, and HIV have become apparent as well. Most of the antiviral drugs can have undesirable side effects, and viruses also grow resistant to the antiviral drugs. Furthermore, with the new emerging infections, more innovative and effective strategies are required to combat against these diseases.

One of the approaches to combat virus resistance and to increase the effectiveness of the antiviral drugs is the use of tailored drug delivery systems, particularly nanoparticular systems such as liposomes, polymeric micro- and nanoparticles, nanogels, and nanofibers [127,128]. Other than its own antiviral activity, chitosan has been a promising material to deliver antiviral drugs both systemically and locally for treatment of numerous viral infections [81,112]. It has been shown to enhance the permeability of the tissue to the drug, increase the contact time of the drug at the application site, target the drug to the desired region, and provide controlled/prolonged drug release. It is also possible to reduce undesirable side effects by means of treatment with nanoparticle systems.

The antiviral activity of chitosan and its derivatives can be associated with its ability to enhance antiviral immune responses, yet the mechanism for its antiviral activity still requires comprehensive research. Recently, He et al. [129] have shown that the chitosan derivative 6-amine-6-deoxidation exerted antiviral activity against Newcastle disease. With increased expression levels of TNF-a and IFN-b, it was suggested chitosan stimulates immune responses and inhibits virus transcription. In another study, it was demonstrated that 3,6-*O*-sulfated chitosan inhibits HPV infection by direct binding to the viral capsid proteins and down-regulates the PI3K/Akt/mTOR pathway in HeLa cells that is linked with autophagy [130].

Chitosan has been investigated mostly for delivery of antiviral drugs. It is noticeable that most of these studies were performed in vitro, with very few in vivo studies. Tenofovir, acyclovir, and imiquimod are among the drugs delivered in these studies. Various dosage forms, such as thermosensitive gels [131], vaginal tablets [132], and injectable nanoparticulate systems [133], were developed, and the permeability, drug release, and biocompatibility of these systems were demonstrated. In all formulations, chitosan and derivatives were found to enhance the permeability of the drug across different mucosae (vagina, nasal, etc.), increase the contact time on the application site, and prolong the drug release. The developed formulations were shown to be compatible.

## 4. Chitosan and Vaccine Adjuvant/Delivery Systems

Vaccines are the most effective way to prevent many infectious diseases. Successful vaccination depends on the ability of vaccines to induce long-term protective immunity [134]. While live-attenuated antigens can induce sufficient immune responses on their own, due to their safety concerns, highly purified vaccine antigens have been developed with high safety while their immunogenicity has decreased. In order to enhance the immune responses, adjuvants are required in vaccine formulations [135]. Aluminum salts are still the most used adjuvant in the licensed vaccines, while there are a limited number of adjuvant systems that are approved with the vaccine formulation. Nevertheless, research is still continuing to develop safer and more efficacious adjuvants [136,137,138,139]. Chitosan has been an attractive material as an adjuvant due to its immunostimulatory activity. Furthermore, its potential utilization as a delivery system makes chitosan promising both as an adjuvant and delivery system for vaccines [140]. The immunostimulatory activity is attributed to its ability to induce innate immune cells to release several cytokines, chemokines, growth factors, and bioactive lipids. In earlier studies, it has been reported that chitosan activates macrophages and induces cytokine secretion from natural killer cells to promote immune response [141,142,143]. In a more detailed study, cGAS-STING and NLRP3 were identified as intracellular signaling pathways for immunostimulatory activity of chitosan [144].

Immunostimulatory activity of chitosan has been shown to be influenced by its physicochemical properties such as molecular weight, deacetylation degree, and solubility [32,143,145,146,147]. In most of the studies, the vaccine systems were prepared using water-soluble chitosan, such as chitosan glutamate. Chitosan derivatives, including trimethylated chitosan with a different degree of quaternization, carboxymethylated chitosan, etc., were also investigated for vaccine delivery [38,41,42,44,146,148].

In a study by Zheng et al. [143], the immunomodulatory activity of different molecular weight chitosans (3 kDa and 50 kDa) was investigated in vitro. Similar responses between different chitosans were obtained in RAW264.7 macrophages, whilst only low molecular weight chitosan was able to induce higher mRNA expression levels of IKKβ, which is an important molecule for NF-κB activation in response to pro-inflammatory stimuli, compared to that of 50 kDa chitosan at the same dose. It was concluded that chitosan activates macrophages in a molecular weight-dependent manner. Recently, Lampe et al. [149] investigated chitosan as an adjuvant at two different molecular weights against influenza A virus (IAV). Both low molecular-weight and high molecular-weight (HMW) CS was found to induce interferon regulatory factor pathway signaling, antigen-presenting cell activation, and cytokine messenger RNA (mRNA) production, with LMW inducing higher mRNA levels at 24 h and HMW elevating mRNA responses at 48 h. It was concluded that both LMW and HMW chitosan show adjuvant activity, while this activity was mediated through different mechanisms based on chitosan molecular weight.

Chitosan and its derivatives have been investigated as vaccine adjuvants/delivery systems against numerous pathogens [140,150]. Delivery systems in different forms (e.g., aqueous dispersion, nano- and microparticles, microneedles, gels, etc.) have been shown to induce both humoral and cellular immune responses [9,140,151,152,153,154]. It has been reported that chitosan increased cellular expansion in local lymph nodes following subcutaneous administration [155]. This is important, especially for systemic delivery, as lymph-node expansion is associated with vaccine efficacy and adaptive immunity, resulting in enhanced vaccination outcomes [156].

In general, particulate chitosan-based systems have been shown to induce the immune system more significantly. Their similar size (<10 μm) to that of the pathogen allows them to be efficiently internalized by the antigen-presenting cells (APCs), including macrophages and dendritic cells (DCs), resulting in enhanced immune responses [157]. Chemical and physical properties of the particulate system significantly influence the uptake by the APCs. In addition to particle size, the surface charge of the vaccine system has also an important impact on stimulation of the immune response. Cationic particles have been shown to be specifically effective for uptake by the APCs. This is attributed to the enhanced binding to the negatively charged cell surface by the positively charged particle, which subsequently increases the internalization. Microparticles (2–3 μm) were effectively taken up by macrophages through receptor-mediated endocytosis and phagocytosis, while nanoparticles, with their size allowing micropinocytosis, uptake of nanoparticles was favored more by dendritic cells when compared to the microparticles [158]. Shape of the particles has been shown to influence the cellular uptake, resulting in higher uptake with spherical shape [138,159,160,161]. Furthermore, the particulate systems provide both in vitro and in vivo stability of the antigen and also enable incorporation with other adjuvants. There are few studies where the chitosan vaccine delivery systems have been tested for vaccination in preclinical and clinical trials [152,162,163,164,165,166].

Chitosan-based vaccine systems can be delivered through various administration routes. In general, these systems are administered systemically via the parenteral route, including subcutaneous (sc), intramuscular (im), and intradermal (id) [140,167]. Intraperitonal (ip) injections are generally applied in laboratory animals.

Vaccines can also be delivered through mucosal surfaces such as oral, nasal, pulmonary, buccal/sublingual, vaginal, etc. [168]. As most infectious agents come into contact with the host at mucosal surfaces, mucosal vaccination is desired to provide both mucosal and systemic immune responses through the common mucosal immune system (CMIS), which links all mucosal surfaces and allows the induction of immune responses at distant sites away from the site of antigen presentation [169]. Stimulation of mucosal immunity can also provide an additional barrier against the invading pathogens and avoid their entry into the body. While the mucosal route offers a non-invasive (needle-free) delivery, vaccination through mucosal membranes requires adjuvants in order to enhance the immunogenicity of the vaccine antigen. It is also important to avoid antigen degradation and to target the antigen to the site of immune function. Hence, an appropriate delivery system is required to provide an enhanced immune response besides a potent adjuvant.

Other than mucosa, skin is the other first line of defence preventing the entry of pathogens into the body, with a unique immune system. Hence, cutaneous immunization (also referred to as skin immunization or transcutaneous immunization) has been approached as a promising alternative to the parenteral route, which provides non-invasive needle-free administration [170,171,172].

## 5. Recent Applications of Chitosan as an Adjuvant and Vaccine Delivery System

Chitosan and its derivatives have been used both as an adjuvant and delivery system for vaccines against numerous viral, bacterial, and fungal diseases. Its adjuvant efficacy has been compared with regard to the type of chitosan used as well as the properties of the delivery system. In most studies, chitosan was used alone, while in other studies it was combined with another adjuvant such as aluminum, etc. In general, the composition of an adjuvant system plays an important role in defining its overall performance besides its physical parameters such as particle size, surface charge, and viscosity. The advantage of chitosan-based systems over other polymers is that they provide both delivery and immunomodulatory properties. Recent advances in understanding of mechanisms involved in innate and adaptive immunity induction allowed to design new adjuvants in a more rational design, specifically induced the pattern recognition receptors (PRRs) [136,173,174]. PRRs are the sensors of the innate immune cells that recognize pathogen-associated molecular patterns (PAMPs), which are molecular motifs associated with pathogens. Upon recognition, activation of innate immunity releases various cytokines or factors that initiate adaptive immunity. Chitosan has been shown to activate the cGAS-STING pathway and the NLRP3 inflammasome, which contains specialized pattern recognition receptors (PRRs) [144]. Recently, Zhao et al. [175] showed that similar to chitosan, nanoparticles prepared with chitosan derivatives *N*-2-hydroxypropyl trimethyl ammonium chloride chitosan and *N*,*O*-carboxymethyl chitosan stimulated the immune responses through the cGAS-STING pathway. Studies reported on carboxymethyl chitosan were not included in this review due to brevity. Readers are referred to our recent review on applications of carboxymethyl chitosan [38].

Our group has developed a combined adjuvant delivery system containing a combination of water-soluble chitosan and outer membrane proteins (porins) of *Salmonella Typhi*, which was reported to activate the innate immune system through TLR2 (toll-like receptor) and TLR4 signals as well as to increase the expression of costimulatory molecules and cytokines on dendritic cells and B cells [176]. The adjuvant activity of this combined system prepared in micro- and nanoparticulate forms was demonstrated in vivo in mice using multistage recombinant antigens (rBAG1 + rGRA1) against *Toxoplasma gondii* (*T. gondii*) infection [161]. We have further compared the adjuvant efficacy of this chitosan-based adjuvant system to that of a combined system based on porins and cationic or anionic liposomes [138]. Both polymeric and liposomal adjuvant systems were shown to provide enhanced immune responses in BALB/c mice following immunization with a model antigen, ovalbumin. Although the IgM levels obtained with liposomal systems were observed to be higher than that with chitosan-based systems, a chitosan-porins-based adjuvant system with micron-size g (5.93 ± 0.03 µm) was found to stimulate higher IgG levels when compared to that of cationic liposomes (383.5 ± 72.5 nm) as well as nano-sized particles (429.9 ± 6.8 nm), indicating long-lasting immunogenicity and protection.

El-Sissi et al. [177] have investigated in vivo the potency of Rift Valley Fever (RVF) inactivated vaccine in the presence of chitosan in different forms, solutions, and nanoparticles (282 ± 110 nm) in comparison to aluminum adjuvant. The highest immune responses were obtained with chitosan nanoparticles loaded with the RVF virus. With chitosan, both humoral and cellular responses were reported to be induced, while with aluminum only the humoral responses were induced. In our previous study, we investigated the aqueous dispersion and nanoparticulate forms of chitosan and its derivatives, *N*-trimethyl chitosan (TMC, polycationic), and mono-*N*-carboxymethyl chitosan (MCC, polyampholytic), loaded with tetanus toxoid antigen, and demonstrated that following nasal immunization, both aqueous dispersion and nanoparticles enhanced mucosal immune responses. The cationic chitosan and TMC nanoparticles induced higher serum IgG titres when compared to those of MCC nanoparticles, which are negatively charged and smaller in size [45]. In another study, TMC was investigated for intradermal vaccine delivery in solution and nanoparticle (200 nm) form [178]. With diphtheria toxoid (DT)-containing TMC formulations, IgG titres and neutralizing antibody titres were shown to be similar to those obtained after subcutaneous injection of DT-Alum. Both TMC solution and TMC nanoparticles were shown to induce dendritic cell maturation and enhance immune responses after intradermal injection.

### 5.1. Systemic Delivery

Chitosan and its derivatives have been investigated as adjuvant/delivery systems for a number of systemically administered vaccines, including influenza [179,180], Hepatitis A [181], *Haemophilus influenzae* type b (Hib) [182], and systemic candidiasis [183]. In a Phase I/IIa clinical trial, the combination of a chitosan-based hydrogel with the Hib vaccine was found to be safe and well tolerated after a single i.m. injection, indicating the potential of chitosan to be used as a safe vaccine carrier [184].

Recent studies have shown that the immunostimulating property of chitosan derivatives is more profound for systemic immunization. Korupalli et al. [185] have incorporated the TMC nanoparticles into a negatively charged hyaluronic acid hydrogel to obtain a depot effect in the injection site and avoid the burst release of the vaccine antigens. The nanocomposite hydrogel system (NPs-Gel) was shown to retain a large proportion of its TMC nanoparticles that are bonded by covalent/electrostatic interactions and extend the release of the encapsulated OVA, enabling their localization at the site of hydrogel injection. The positively charged TMC nanoparticles were observed to be effectively internalized by dendritic cells. The TMC nanoparticles, which do not have any specific interactions with the hydrogel network, were shown to be released rapidly and internalized by the neighboring immune cells, providing a priming dose, while those retained inside the gel were ingested by the recruited and concentrated immune cells over time, acting as a booster dose, eliciting high titers of OVA-specific antibody responses.

In another study, the TMC-based nanoparticles were used for systemic delivery of the dengue vaccine [186]. After immunization in BALB/c mice, enhanced cellular uptake and strong induction of humoral and cellular immune responses with mixed Th1/Th2 mixed immunity were obtained. Similarly, TMC nanoparticles incorporated with UV-inactivated Dengue virus-2 (DENGV2) were shown to be effectively taken up by the human monocyte-derived dendritic cells, inducing the maturation of dendritic cells [187]. High humoral (IgG, IgG1, and IgG2a levels) and cellular (CD8 and CD4 T) immune responses and neutralizing activity were obtained after intraperitoneal immunization of mice. In another study of the same group [188], a bivalent form of nanoparticle-based dengue vaccine was developed using C-terminus truncated non-structural protein 1 (NS1_1–279_) and envelope domain III (EDIII) of DENV-2 encapsulated in the nanocarriers, *N*,*N*,*N*-trimethyl chitosan nanoparticles (TMC NPs). It was concluded that following intraperitoneal immunization of mice, NS1 + EDIII TMC NPs induced protective responses that can not only neutralize infectious DENV-2 but also eliminate DENV-2-infected cells.

Recently, Li et al. [189] have developed a novel composite system based on a chitosan derivative that was named analogous hyaluronic acid chitosan (AHAC), which resembled hyaluronic acid in structure. A thermosensitive nanoparticle-hydrogel (AHACNP-HG) composite consisting of AHAC nanoparticles (AHAC-NPs) and AHAC hydrogel (AHAC-HG) was loaded with *T. gondii* microneme protein 6 and rhoptry protein 18, and following four subcutaneous immunizations in total at a 2-week interval, mixed Th1/Th2 cellular immune responses were obtained and a prolonged survival time after intraperitoneal challenge with *T. gondii* tachyzoite compared to that of the control group.

Animals vaccinated with the various chitosan-based vaccines were also shown to be protected against various pathogens. Zhou et al. [190] evaluated in vivo the adjuvant activity of a water-soluble derivative of chitosan, *N*-2-hydroxypropyl trimethyl ammonium chloride chitosan (*N*-2-HACC), for delivery of porcine parvovirus vaccine (PPV). *N*-2-HACC was observed to induce higher and longer-lasting HI antibodies and cellular immune responses compared to the commercial vaccine. Immunization of PPV/*N*-2-HACC was shown to protect the sows challenged with the homologous PPV-H strain, mainly by enhancing humoral immunity rather than cellular immunity. In another study with HACC, the adjuvant properties of chitosan base and two chitosan derivatives (HACC and sulfated chitosan) in nanoparticle form incorporated with inactivated Newcastle disease vaccine (NDV) were investigated in comparison to the commercial vaccine in chickens following subcutaneous immunization [191]. It was demonstrated that the humoral immunity levels in chitosan containing groups were lower than those in commercial vaccines, while higher cellular immunity levels were obtained. Moreover, the protective effects of the chitosan-containing particles were found to be comparable to those of a commercial vaccine. Chuang et al. [192] developed nanoparticulate delivery systems for the anthrax vaccine using HACC and fucoidan (FUC), which has anticoagulant, antivirus, anti-oxidant, anti-inflammatory, antitumor, and immunomodulating activity. Positively and negatively surface-charged FUC-HTCC NPs were prepared via polyelectrolyte complexation by varying the mass ratio of FUC and HTCC. Negatively charged particles showed higher cellular uptake in dendritic cell lines, while higher antibody responses and protection were obtained with positively charged particles. Similarly, the porcine parvovirus V2 subunit (PPV/VP2) vaccine in HACC solution was shown to induce high and long-lasting antibody levels with 100% protective efficacy compared to the commercial vaccine against porcine parvovirus infection in sows [193].

Aside from being used alone, chitosan has been combined with other compounds to enhance immune responses. Soares et al. [194] have combined chitosan with β-glucan biopolymers in the same particle, preferably with surface β-glucan localization to simulate the cell wall of some pathogens and to stimulate the immune cells expressing the Dectin-1 receptor. Following subcutaneous immunization in mice, β-glucan-containing particles were shown to induce 16-fold higher IgG antibody titers against hepatitis B surface antigen (HBsAg) compared to that of chitosan nanoparticles alone.

### 5.2. Mucosal Delivery

For mucosal immunization, chitosan and its derivatives have been applied mainly to nasal or oral (intestinal) mucosa.

While oral vaccination is painless, safe, and easy for all ages, the harsh acidic environment in the gastrointestinal tract and the presence of proteolytic enzymes result in degradation of the antigen. Additionally, antigens are poorly taken up by the immune system, and high repeated doses of vaccine are required for efficient immunization [195]. Hence, a vaccine system that ensures antigen stability, shows mucoadhesive properties, and provides efficient targeting of antigen to immune cells is required for oral immunization. Chitosan has been successfully applied as a delivery system, providing adhesion to the mucosa, protection of the antigen from degradation, and facilitating immune cell targeting, which results in enhanced immune responses [151,152,196,197,198,199,200]. Furthermore, it has been used for coating the particulate systems based on different polymers [201,202]. Due to its cationic nature and mucoadhesive property, it can protect the antigen form degradation and also prolong the contact time of the system, thereby enhancing the antigen uptake. Recently, chitosan-coated bovine serum albumin-loaded mesoporous silica nanoparticles (345 ± 60 nm) were shown to induce IgG immune responses following oral administration to mice [203]. These results confirmed that chitosan coating efficiently protects the antigen from the gastrointestinal environment and promotes antigen uptake by M cells, generating both mucosal and systemic immune responses. Interestingly, higher IgA levels were observed in the presence of chitosan, which indicated the adjuvant activity of chitosan as well.

In a recent study, chitosan nanoparticles encapsulating filarial recombinant antigens of *Brugia malayi* (TRX and ALT-2) against lymphatic filariasis, a parasitic disease affecting the lives of millions of people in tropical regions, were evaluated following oral or nasal vaccination in mice [197]. High antibody levels were obtained with both routes, with the highest humoral responses and more balanced Th1/Th2 antibody isotype responses with oral. On the other hand, higher IgG1 antibody titers and Th2-shifted immune responses were obtained with the nasal route.

An oral vaccine using the truncated capsid protein p146 (aa460–605) against Hepatitis E based on chitosan nanoparticles (200–300 nm) was developed [204]. The chitosan-based vaccine system was shown to avoid antigen degradation and provide systemic and mucosal immune responses compared to the purified antigen alone. Additionally, higher expression levels and mRNA transcription of IL-4 in spleen cells were obtained with chitosan nanoparticles, indicating the activation of Th2-mediated cellular immune response.

Gao et al. [205] evaluated the potential of composite nanoparticles (219 ± 13.7 nm) based on using oppositely charged chitosan derivatives, N-2-hydroxypropyl trimethyl ammonium chloride chitosan (cationic)/*N*,*O*-carboxymethyl chitosan (anionic), using ovalbumin as the model antigen. Humoral and cellular immune responses were obtained with both intramuscular and oral immunization, whereas IgA was elicited only in orally immunized mice, confirming the immunostimulatory activity of chitosan derivatives through oral delivery.

Another approach investigated to enhance the performance of chitosan-based systems for vaccination includes surface modification with other polymers such as polyethylene glycol (PEG), poloxamer, Eudragit^®^, and sodium alginate [206,207]. The type and physicochemical characteristics of the materials used for coating have been shown to have an impact on the success of the vaccine delivery. In a recent study, OVA-loaded chitosan nanoparticles were coated with PEG or sodium alginate (Alg) [208]. The hydrophilic polymer PEG was selected due to its ability to form a shielding layer around chitosan nanoparticles, thereby enhancing its stability in the gastrointestinal system. Sodium alginate was used for its mucoadhesive property. The Alg and PEG surface modifications of chitosan nanoparticles were shown to improve the stability of the system in both simulated gastric and intestinal fluids with improved mucoadhesive properties, highest with Alg. It was reported that the surface charge, hydrophobicity, size, and zeta potential of the particles had an impact on their stability after coating. Similarly, other studies based on Alg coating of the chitosan nanoparticles were reported to enhance the stability of the antigens, such as lipopolysaccharides derived from *Vibrio Cholerae* [209] and Hepatitis B [198] for oral delivery.

Buffa et al. [210] have investigated the delivery of chitosan and various TLR ligands by different administration routes such as sublingual, intranasal, intravaginal, and subcutaneous in a murine model. Systemic and mucosal immune responses were evaluated against HIV-1 CN54 gp140 and tetanus toxoid. Intranasal immunization was shown to provide the best balance between systemic and mucosal responses, with most of the adjuvants evaluated. The IgG levels with intranasal were found to be equivalent to those of subcutaneous and better than sublingual immunization. For IgA levels, intranasal was equivalent to or better than sublingual, and both were better than subcutaneous immunization.

The efficacy of methylglycol chitosan (MGC) and a synthetic toll-like receptor 4 agonist (CRX-601), alone or in combination, was investigated for improving systemic and mucosal immune responses against a monovalent detergent-split flu virus vaccine delivered sublingually [211]. Sublingual immunization with either methylchitosan or CRX-601 vaccine formulation was shown to provide higher serum IgG and mucosal IgA titers compared to non-adjuvanted vaccines and were equivalent to or greater than that of intramuscular vaccination. Chitosan-based systems have been investigated for oral vaccination as well as for veterinary vaccines such as fish vaccination against *Vibrio anguillarum* [212,213] and Koi herpesvirus [214], fowl typhoid [215], and porcine epidemic diarrhea virus [216].

As nasally administered vaccines have been shown to provide effective immunostimulation, most of the mucosal immunization studies with chitosan-based systems have focused on the nasal route [217]. Several animal studies have been carried out against influenza, pertussis, and diphtheria vaccines with satisfactory results. After nasal administration of the chitosan-based vaccines, significant serum IgG responses were induced similar to and secretory IgA levels higher than those induced by systemic administration of the vaccine. Chitosan alone, its methylated derivatives have been widely investigated for this purpose [38,218,219]. The potential of chitosan-based systems as adjuvants and delivery systems has been developed, which also protect the antigen from enzymatic degradation in nasal fluid and extend its retention time in the nasal cavity [42,140,220,221]. Combination of chitosan with other polymers, adjuvants, or other materials was shown to enhance the immune responses against various antigens [220,222,223,224,225].

Particularly, cationic water-soluble chitosan and its derivatives have been shown to increase the nasal residence time of whole inactivated influenza virus when a nanoparticle formulation was administered intranasally [219]. In our earlier studies, we have shown that cationic chitosan nanoparticles (397.8 ± 7.8 nm) and *N*-trimethyl chitosan nanoparticles (306.0 ± 2.4 nm) loaded with tetanus toxoid induced higher serum IgG titres when compared to that of negatively charged mono-*N*-carboxymethyl chitosan nanoparticles (90.9 ± 0.8 nm) following nasal immunization [45]. The results emphasized the significant role of surface charge and particle size in the uptake of vaccine formulations by macrophage J774A.1 cells and induction of mucosal and systemic immune responses.

Nevagi et al. [226] have reported a nanoparticulate system based on ionic interactions between cationic trimethyl chitosan (TMC) and a peptide antigen coupled with synthetically defined anionic α-poly-(l-glutamic acid) (PGA). The antigen, possessing a conserved B-cell epitope derived from the group A streptococcus (GAS) pathogen and a universal T-helper epitope, was conjugated to PGA using cycloaddition reaction. Following nasal immunization with the nanoparticles in mice, both systemic and mucosal immunity was observed at low doses. Additionally, nanoparticles provided protection to vaccinated mice against group A streptococci infection. In another study, sodium alginate (Alg)-coated chitosan and TMC nanoparticles loaded with inactivated PR8 influenza virus were prepared by direct coating of the virus with chitosan or TMC, and their adjuvant activity was evaluated following nasal immunization in BALB/c mice. PR8-chitosan formulation was found to elicit higher IgG2a and IgG1 antibody titers compared to that of PR8-TMC. Alg coating (PR8-chitosan-Alg) was found to significantly decrease the antibody titers, and lower immune responses were induced compared to PR8-TMC-Alg formulation. A significantly higher IgG2a/IgG1 ratio was obtained with the PR8-TMC-Alg formulation, indicating a Th1-type immune response. It was reported that the PR8-TMC-Alg formulation would be suitable for intranasal antigen delivery systems [224].

Najminejad et al. [227] have encapsulated two *Bordetella pertussis* antigens, Pertussis toxoid (PTd) and filamentous hemagglutinin (FHA), into *N*-trimethyl chitosan (TMC) nanoparticulate systems. Nasal immunization with the PTd-FHA-loaded TMC nanoparticles was shown to induce both systemic and local mucosal immune responses, which is expected to improve the efficacy of pertussis prevention through respiratory tract transmission.

Pawar et al. [228] have investigated chitosan and glycol chitosan as mucoadhesive and cationic coating material for nasal vaccine delivery of hepatitis B surface antigen (HBsAg). Higher local and systemic uptake, higher nasal retention, and systemic and mucosal antibody responses against Hepatitis B surface antigen were obtained with glycol chitosan-coated particles compared to chitosan-coated particles. Rose et al. [229] have coated the lipid-polymer hybrid nanoparticles (LPNs) with the glycol chitosan for its mucoadhesive property. Increased antigen-specific mucosal immune responses were induced in the lungs and the genital tract with the optimized GC-coated LPN adjuvant upon nasal immunization of mice with the recombinant Ct fusion antigen CTH522 against *Chlamydia trachomatis*.

Korupalli et al. [185] incorporated the TMC nanoparticles into a negatively charged hydrogel system to obtain a depot effect in the injection site and avoid the burst release of the vaccine antigens. The nanocomposite hydrogel system (NPs-Gel) was shown to retain a large proportion of its TMC nanoparticles that are bonded by covalent/electrostatic interactions and extend the release of the encapsulated OVA, enabling their localization at the site of hydrogel injection. The positively charged TMC nanoparticles were observed to be effectively internalized by dendritic cells. The TMC nanoparticles, which do not have any specific interactions with the hydrogel network, were shown to be released rapidly and internalized by the neighboring immune cells, providing a priming dose, while those retained inside the gel were ingested by the recruited and concentrated immune cells over time, acting as a booster dose, eliciting high titers of OVA-specific antibody responses.

Thiolated chitosan with enhanced mucoadhesive properties has also been investigated for vaccine delivery [230]. In a recent study, virus-like particles of influenza A virus (IAV) were encapsulated in thiolated chitosan [40]. The thiolation degree of chitosan was reported to affect the physicochemical properties, cytotoxicity, blood compatibility, as well as mucoadhesion and immune response levels. Mucin adsorption of thiolated chitosan nanoparticles containing high thiol group content (511 and 764 μmol/g) was found to be 2.2-fold higher than that of unmodified chitosan nanoparticles. Furthermore, thiolated chitosan nanoparticles were shown to enhance immune responses and also provide complete protection against swine and avian IAV infection, showing cross-protection against different subtypes of IAV strains.

In our recent study, aminated and aminated plus thiolated chitosan were investigated for nasal immunization using bovine serum albumin (BSA) as a model antigen [41]. Higher IgG levels were obtained with the modified chitosan nanoparticles, which induced higher levels of systemic antibodies (IgG, IgG1, and IgG2a), resulting in stimulation of mixed Th1/Th2 immune responses. Moreover, sIgAs were detected in vaginal washes, suggesting stimulation of the common mucosal immune system.

More recently, novel chitosan-curdlan composite nanoparticles (211.0 ± 3.6 nm) loaded with BSA were prepared by the polyelectrolyte complexation method using carboxymethyl curdlan as a polyanionic polymer and chitosan chloride or *N*-trimethyl chitosan as polycationic polymers [42]. In vivo studies in mice indicated that nasal and subcutaneous administration of composite nanoparticles could provide long-term humoral and cellular immunity. Elevated sIgA levels after nasal administration of the nanoparticles showed that mucosal immunity was successfully stimulated.

Additional adjuvants combined with chitosan particles have also been evaluated for nasal immunization. For example, when the PPE17 antigen of *Mycobacterium tuberculosis* and CpG was loaded onto alginate-coated chitosan nanoparticles with a size of 427 nm and a zeta potential of −37 mV, Th1 immune responses were enhanced [222].

The pulmonary route also provides a noninvasive, needle-free route to deliver vaccines. Like other mucosal routes, pulmonary vaccination can efficiently induce both systemic and mucosal immune responses [231]. Recently, the efficacy of chitosan nanoparticles loaded with the model antigen OVA co-administrated with the STING agonist, bis-(3′,5′)-cyclic dimeric adenosine monophosphate (c-di-AMP), was evaluated in mice following pulmonary delivery [232]. In addition to enhanced antigen-specific IgG titers, a strong Th1/Th17 response characterized by high secretion of IFN-γ, IL-2, and IL-17, as well as induction of CD8^+^ T cells, was obtained. This system was suggested as a promising technology platform for delivery of vaccines against respiratory pathogens (e.g., influenza or RSV). Recent studies on chitosan-based pulmonary delivery systems for vaccines as well as drugs for COVID-19 will be reviewed separately in the following section.

In Table 4, recent studies focusing on the intranasal vaccination using chitosan-based formulations are summarized, focusing on the antigen delivered, chitosan type used, delivery system developed, and its characteristics, as well as results obtained from in vitro and in vivo studies.

**Table 4:** Recent studies on chitosan-based vaccine delivery systems for nasal application.

Antigen	Chitosan Type	Delivery System	Additional Adjuvant	In Vitro Studies	In Vivo Studies	Results	Ref.
Influenza A virus recombinant protein as NP-Cap-M2e protein self-assembles into virus-like particles (NMC VLPs)	Thiolated chitosan synthesized using chitosan (Mw: 50–190 kDa)	Thiolated chitosan (TCS) NPs encapsulating VLPs (196 ± 3.14 nm)		–Cytotoxicity in RAW264.7 and DC2.4 cells –Hemolysis assay in chicken blood erythrocytes –Phagocytosis assay in RAW264.7 and DC2.4 cells	Immunization in female mice Dose: NCM VLPs in TCS NPs (20 μg) NCM VLPs alone (6.8 μg) Recombinant proteins alone (1.65 μg 3M2e + 0.68 μg NPep) Booster: Days 21 and 42 Challenge: –10 × LD_50_ of A/Puerto Rico/8/1934 (H1N1) –10 × LD_50_ of A/swine/Henan/1/2010 (H3N2) and 50 μL 10^9^TCID_50_/mL of A/chicken/ Guangzhou/GZ/2005 (H9N2) viruses –5 × LD_50_ of A/Puerto Rico/8/1934 (H1N1) virus –Humoral immune responses in sera and lung lavage fluid –IFN-γ and IL-4 secretion levels –IAV titers	–Enhanced humoral, cellular, and mucosal immunity –No toxicity –The degree of thiolation affects the immune responses –Increased phagosytosis –Enhanced systemic and mucosal immune responses (Th1-biased) –Increased IFN-γ and IL-4 levels –Reduced viral replication and prevention of severe lung damage –Complete protection against swine and avian IAV infection (CD8+ cells playing an important role) –Cross-protection against different subtypes of IAV strains	[40]
PPE17 recombinant protein expressed in *E. coli* BL2 against tuberculosis	Chitosan (8 cP viscosity, DD: 98%)	Alginate-coated chitosan NPs (427 nm)	ISCOMATRIX		Immunization in BALB/c mice (6–8 weeks old) Dose: 6 µg Booster: Days 14 and 28 –IgG1, IgG2a titers and IFN-γ, IL-4, IL-17, and TGFβ cytokine levels –s.c. application for comparison	–Higher levels of IgG2a and IgG1 in the presence of adjuvant after i.n. and s.c. administration –Stronger IFN-γ following coadministration of ISCOMATRIX and NPs after i.n. administration compared to s.c. –Higher IL-17 and IL-4 responses with s.c. administration	[233]
BSA	Chitosan chloride and *N*-trimethyl chitosan (TMC) synthesized using chitosan (9 cP)	Chitosan chloride-carboxymethyl curdlan composite NPs (211.0 ± 3.6 and 239.0 ± 1.6 nm) For comparison: –TMC aqueous dispersions –Carboxymethyl curdlan aqueous dispersion		–Cytotoxicity in A549 and Calu-3 cells –Cellular uptake in J774A.1 cells	Immunization in female BALB/c mice (6–8 weeks) Dose: 20 μg Booster: Day 14 –Humoral and cellular immune responses s.c. administration for comparison BSA-alum (s.c.) BSA-CpG solution (i.n.)	–Higher cell viability with NP compared to dispersions –Complete particle uptake in 2 h –Enhanced immune responses on Day 21 –Stimulation of humoral and cellular immune responses –High sIgA levels in vaginal washes after i.n. immuniziation with NPs –Higher IL-4, IL-6, and IL-10 levels with NPs (s.c. and i.n.) –Highest IL-2 and IFN-γ levels obtained with i.n. administered NPs	[42]
BSA	*N*-2-hydroxypropyltimehyl ammonium chloride chitosan and carboxymethyl chitosan synthesized in-house (Mw and DD not stated)	*N*-2-hydroxypropyl trimethyl ammonium chloride chitosan/*N,O*-carboxymethyl chitosan NPs		–Cytotoxicity in 293T cells –Cell apoptosis in 293T cells –Cellular uptake in DC2.4	Immunization in female BALB/c (6−8 weeks) mice Dose: 30 μL of 3 μg/μL Booster: days 10, 20 and 30 –Safety testing –IgG and its subtype levels in serum and IgA in the genital tract lavage fluid and nasal cavity lavage fluid –Cytokine levels (IL-4 and IFN-γ) –Splenocyte proliferation –Activation of the TLR4/NF-κB signaling pathway in RAW264.7 cells	–Higher safety –Significant cellular uptake –Long-time retention on nasal mucosa –Higher humoral responses (Th1/Th2 immunity) –Local and distant mucosal immune responses –Enhanced cellular immune responses –Activation of the TLR4/NF-κB signaling pathway	[234]
*Haemophilus influenzae* recombinant outer membrane protein P6	Mannose-modified chitosan synthesized using chitosan (low Mw, deacetylation 75–85%)	Mannose-modified chitosan microspheres (MCM) (590.4 ± 16.2 nm) Chitosan-microspheres (463.7 ± 15.1 nm)			Immunization in 6-week-old female specific pathogen-free (SPF) BALB/c mice Dose: 20 μg Booster: Days 14 and 28 –P6-specific systemic and mucosal immune responses (IgA and IgG) –Th1-type, Th-2-type, and Th-17-type cytokines –CD3+, CD4+, and CD8+ T lymphocyte subpopulations –Challenge: NTHi in a bacterial suspension (1×10^8^ CFU/mL)	–MCM improved humoral (IgG) and mucosal (IgA) immunity compared to chitosan microspheres –MCM enhanced cellular immunity and also triggered a mixed Th1/Th2-type immune response –MCM could promote lymphocyte proliferation –MCM induced MHC class I and II-restricted antigen presentation and generated CD4+-mediated and CD8+ mediated immune responses –P6-MCM reduced inflammation in nasal mucosa and lung and showed strong protection against NTHi infection	[235]
PPE17 protein of *Mycobacterium tuberculosis* expressed in *E. coli* BL21	Chitosan (8 cP viscosity, DD: 98%)	Alginate-coated chitosan NPs (427 ± 8.6 nm) Chitosan NPs (278 ± 4.5 nm)	CpG		Immunization in male BALB/C mice (6 to 8 weeks old) Dose: 6 μg Booster: Days 14 and 28 –Serum IgG1 and IgG2a –IFN-γ, IL-4, IL-17, and TGF-β levels –s.c. administration for comparison	–Strong humoral immune responses –Increased IFN- γ, IL-4, and TGF-β levels –No detectable IgG2a	[222]
OVA, Influenza virus nucleoprotein peptide	Chitosan (medium Mw: 400,000)	Chitosan-hydrogel with poloxamer 188 and poloxamer 407 Chitosan solution (non-gel formulation)	LPS (Sigma)	In vitro activation of OT-I cells	Immunization in mice *OVA immunization* Dose: 30 µg Challenge: i.n. with 3 µg of influenza virus nucleoprotein peptide –T cell priming 3 days after immunization –Deposition of resident memory T cells 30 days after immunization –Immune responses in serum, nasal lavage fluid, and bronchial alveolar lavage fluid *Influenza virus nucleoprotein peptide immunization* Priming with chitosan solution (i.p. administration) Booster: with chitosan hydrogel (i.n. administration) on days 7 and 14 using chitosan hydrogel Challenge: 28 days after the final booster with 104.5 PFU of X31(H3N2) –Humoral and cellular responses	–Retention of most OVA within the nasal tissue when incorporated into chitosan-hydrogel and promotion of T cell priming in LNs –6.7-fold more antigen-positive cells in nasal tissue of mice receiving chitosan-hydrogel vaccine compared to non-gel formulation, indicating that increased viscosity resulted in longer retention times in the nasal cavity, which showed CTL priming to the LN draining the upper airways and boosted the development of local nasal Trm CD8+ T cells, whereas accumulation of antigen within lower airways was seen with non-gel formulation, indicating that viscosity of formulation effects retention of antigen at different sites –Generation of equivalent levels of anti-OVA IgG antibodies by chitosan-based vaccines in serum on day 14 post immunization –Mice immunized with a chitosan-hydrogel vaccine loaded with influenza virus peptides developed a 10-fold more influenza-specific CD8+ nasal Trm that provided high protection against influenza challenge	[145]
*Brucella abortus* recombinant proteins (rMdh, rOMP 10 and 19)	Chitosan (Mw is not stated; DD: 82.4%)	Chitosan NPs: rMdh-CS NPs: (475.4 ± 124.5 nm) rOMP 10-CS NPs (360.8 ± 84.5 nm) rOMP 19-CS NPs (439.5 ± 89.0 nm) rCocktail-NPs (rOmp10, rOmp19, and rMdh at 1:1:1 ratio)			Immunization in 6-week-old female BALB/c mice Dose: 30 μg Booster: Days 14 and 28 –IgA, IgG, and its subtypes and cytokine levels	–Increased IFN-γ and IL-4 levels with a mixed Th1-Th2 response –Increased IgA levels with a Th2-polarized immune response following immunization with rCocktail-NPs	[236]
Inactivated Swine Influenza virus (H1N2- OH10) (KAg)	Chitosan (Mw 50–190 kDa; DD: 75–85%)	Chitosan NPs (KAg-CS NPs) (141 nm) KAg-CS+poly(I:C) NPs (411 nm)	Poly(I:C)		Immunization in 5-week-old cesarean-delivered colostrum-derived SwIV antibody free piglets Dose: 10^7^ TCID_50_ Booster: Day 21 Challenge: i.n. and intratracheal with SW/OH/24366/2007 (H1N1-OH7) influenza virus on day 35 –HI assay –Cytokine gene expressions Commercial product administration for comparison (FluSure XP^®^)	–Higher HI titers with KAg-CS+poly(I:C) NPs –Higher Th1 (IFN-γ, IL-6, and IL-2) and Th2 (IL-10 and IL-13) levels compare to the commercial product –No IgA production –Similar reduction in virus load	[237]
MxiH recombinant antigen of *Shigella flexneri* produced in *E. coli* BL21	Chitosan (Mw: 50–190 kDa; DD: 75–85%)	Chitosan NPs (100 nm) MxiH-Freund’s adjuvant (for comparison)			Immunization in 21-day-old male BALB/c mice Dose: Not stated Boost: On days 20 and 55 Challenge: i.n. with *S. flexneri* serotype 1a on day 60 –Determination of IgA, IgG, and IL-4, IFN-γ cytokines	–Enhanced and similar IgG and IgA levels with NPs and Freund’s adjuvant –Higher IFN-γ levels with NPs compared to Freund’s adjuvant –Higher IL-4 levels with NPs compared to CS and MxiH alone –Highest survival rate with NPs (60%) immunized mice compared to Freund’s adjuvant (50%)	[223]
*Acinetobacter* *baumannii* biofilm-associated protein (bap)	Chitosan (Mw: 190–310 kDa; DD: 75–85%)	Chitosan NP (1080 nm) Bap-Freund’s adjuvant (for comparison)			Immunization in 4-to 6-week-old female BALB/c mice Dose: 20 μg Booster: Days 14 and 28 Challenge: i.n. with *A. baumannii* –IgA and IgG levels	–Higher IgG and IgA levels –100% survival	[238]
Group A streptococcus lipopeptides (LP1, LP2, and LP3)	TMC synthesized using chitosan (Mw: 50–190 kDa; DD: 75–85%)	Lipopeptides-TMC NPs: LP1-TMC NPs: (424 ± 19 nm) LP2-TMC NPs: (198 ± 16 nm) LP3-TMC NPs: (126 ± 14 nm)		–DCs and macrophage activation by determination of costimulatory molecule expression on splenic cells from C57/BL6 mice	Immunization in 6-week-old female C57BL/6J mice Dose: 30 μg Booster: Days 21 and 42 –IgA, IgG, and its subtype levels –Indirect bactericidal assay (with sera of immunized mice against GAS strains)	–Higher expression of costimulatory molecules –Highest IgG titers with LP3-TMC NPs following priming and first booster –Increased IgG titers with LP1-TMC NPs and LP3 following 2nd booster –No detectable IgA titers –Highest opsonization with LP3-TMC NPs	[239]
BSA	aCS and atCS synthesized using chitosan (Mw: 50–190 kDa; DD: 75–85%)	Modified chitosan NPs: (125.2 ± 5.5 nm) Chitosan NPs (110.0 ± 3.3 nm)		–Mucoadhesion –Cytotoxicity in Calu-3 and A549 cells	Immunization in 6–8-week-old female BALB/c mice Dose: 20 μg Booster: Day 14 –Determination of IgA, IgG and its subtypes levels and cytokine levels –For comparison Free BSA solution (i.n. and s.c.) BSA-alum (s.c.) BSA-CpG solution (i.n.)	–Concentration-dependent cytotoxicity –Higher IgG and IgA titers and cytokine expression –Induction of Th1-related cytokine levels and Th2-antibody levels with i.n. immunization –Induction of Th2-related cytokine levels and Th1-based antibody levels with s.c. immunization of NPs indicating mixed Th1/Th2 immune responses	[41]
Cocktail antigens of *Brucella* *abortus* 544 (sodC, OMP19, BLS, and PrpA)	Chitosan (Mw and DD are not stated)	Chitosan NPs (size not stated)	LPS of *Brucella abortus 544*		Immunization in 5-week-old female BALB/c mice Dose: 5 μg LPS; 25 μg each antigen (sodC, OMP19, BLS, and PrpA) Booster: Day 14 Challenge: i.n. with *B. abortus 544* on day 30 –IgA, IgG, and IgG subtypes and cytokine levels –Splenocyte cell proliferation	–Higher IgG and mucosal IgA levels –Higher levels of IFN-γ and T cell proliferation –Decreased bacterial load in lungs and spleen –No significant effect with LPS	[240]
Human influenza A/Puerto Rico/8/1934 (H1N1) virus antigen (PR8)	Chitosan and TMC synthesized using chitosan	Alginate-coated and uncoated NPs: CS NPs (380.6 nm) TMC NPs (318.2 nm) Alginate-coated CS NPs (471.1 nm) Alginate-coated TMC NPs (453nm)			Immunization in female BALB/c mice Dose: 15 μg Booster: Days 14 and 28 –IgG and its subtype levels	–Enhanced IgG levels –Highest IgG1 levels with mixed Th1/Th2 immune response after vaccination with PR8-loaded CS NPs –Highest IgG2a levels indicating the Th1-based immune response after vaccination with alginate-coated PR8-loaded TMC NPs	[224]
siRNA sequence against influenza nucleoprotein	Chitosan (Mw: 400 kDa; DD: 84.7%)	Chitosan/siRNA/ nanoparticle complex (278 nm)		–Cell transfection of siRNA and knockdown of EGP in Vero cells	Immunization in BALB/c mice Dose: 30 μL of chitosan/siRNA containing 50 nM siRNA –Challenge: i.n. delivery of a LD of influenza (PR8) virus	–Enhanced transfection and efficient knockdown –Significant inhibition effect on influenza virus replication –>50% protection	[241]

*Acinetobacter baumannii: A. baumannii*; aCS: aminated chitosan; atCS: aminated plus thiolated chitosan; BSA: bovine serum albumin; CpG: cytosine-phosphate-guanine; CS: chitosan; CTL: cytotoxic T lymphocytes; DCs: dendritic cells; DD: deacetylation degree; *E. coli: Escherichia coli*; HI: hemagglutination inhibition; i.n.: intranasal; i.p.: intraperitoneal; IAV: influenza A virus; kDa: kilodalton; LD: lethal dose; LNs: lymph nodes; LP: lipopeptides; DC: dendritic cell; LPS: lipopolysaccharides; MCM: mannose-modified chitosan micro-spheres; mL: milliliter; Mw: molecular weight; nm: nanometer; NP: nanoparticle; NTHi: nontypeable *Haemophilus influenzae*; OVA: ovalbumin; PFU: plaque forming units; rMdh: recombinant malate dehydrogenase; rOmp 10: recombinant outer membrane proteins 10; rOmp 19: recombinant outer membrane proteins 19; *S. flexneri: Shigella flexneri*; s.c.: subcutaneous; SwIV: swine influenza virus; TCID: tissue culture infectious dose; TMC: N-trimethyl chitosan; VLP: virus-like particle; μg: microgram; μL: microliter.

#### Oral Delivery

The presence of immune cells in the oral-pharyngeal mucosa [242] and intestinal mucosa, which consists mainly of gut-associated lymphoid tissues (GALTs) and mesenteric lymph nodes [200], makes oral (gastrointestinal, buccal, and sublingual) delivery of vaccines an attractive alternative for non-invasive immunization. To date, oral liquid vaccine formulations against polio, typhoid, rotavirus, and cholera and sublingual sprays against recurrent urinary tract infections have gained market authorization [243]. While oral vaccination is a painless, safe, and easy way to administer vaccines for all ages, the harsh acidic environment in the gastrointestinal tract and the presence of proteolytic enzymes hinder antigen absorption and restrict the efficacy of particularly recombinant protein antigens. Additionally, antigens are poorly recognized by the immune system, and high repeated doses of vaccine are required for efficient immunization [195]. An ideal oral vaccine should be able to ensure antigen stability, show mucoadhesive properties, and provide efficient targeting of antigen to immune cells. Considering this, incorporation of antigens into chitosan-based particulate systems seems to provide an excellent adjuvant/delivery system that can protect the antigen from degradation, can adhere to the mucous membrane, and can facilitate immune cell-targeting, consecutively enhancing immune responses. Thus, chitosan has been explored for oral vaccination with different protein and nucleic acid antigens in a vast number of studies [151,152,196,197,198,199,200].

Chitosan has been utilized for coating the particulate systems such as liposomes [201] and polymeric PLGA nanoparticles [202] to modify the surface characteristics of the particles, thereby improving the stability of the antigen. It was also used as a mucoadhesive enhancer due to its cationic charge that facilitates prolonged contact time with mucosal surfaces. Recently, chitosan-coated bovine serum albumin-loaded mesoporous silica nanoparticles of 345 ± 60 nm size and 18.28 ± 0.71 mV surface charge were able to induce an IgG immune response in the serum of mice following oral administration [203]. The results indicated that chitosan-coated mesoporous nanoparticles could efficiently protect the antigen in the gastrointestinal tract and promote antigen uptake by M cells. Moreover, systemic and mucosal immune responses were successfully generated. Interestingly, IgA titer levels were higher with chitosan-coated silica nanoparticles than uncoated ones or BSA solution, suggesting the benefits of chitosan coating for stimulation of mucosal immunity.

In a recent study, the potential of chitosan nanoparticles encapsulating filarial antigens TRX and ALT-2 separately to provide protection against Lymphatic filariasis, a parasitic disease affecting the lives of millions of people in tropical regions, was evaluated following oral or nasal vaccination in mice, and both mucosal immunization routes were compared. While both nasal and oral routes exhibited a good antibody profile, orally delivered vaccines could stimulate higher humoral responses and more balanced Th1/Th2 antibody isotype responses. On the other hand, high IgG1 antibody titers and Th2-shifted immune responses were seen for the nasal route. The study suggested that chitosan nanoparticles could be a promising adjuvant/delivery system, particularly for oral vaccination with filarial recombinant antigens of *Brugia malayi* [197].

An oral vaccine candidate based on chitosan was developed against Hepatitis E, while only one vaccine has been commercialized only regionally (Hecolin^®^). The chitosan nanoparticles (200–300 nm) showed high encapsulation efficiency and loading capacity for the novel recombinant truncated capsid protein p146 used as an antigen and demonstrated a sustained release pattern [204]. In addition to avoidance of degradation in the gastrointestinal tract, stronger mucosal, humoral, and cellular immune responses were obtained with p146-loaded nanoparticles. Higher expression levels and mRNA transcription of IL-4 in spleen cells were detected, indicating the activation of Th2-mediated cellular immune response.

Composite nanoparticles developed using chitosan derivatives with opposite charges for oral immunization have been shown to be advantageous compared to the parenteral route. Gao et al. [205] evaluated the potential of *N*-2-Hydroxypropyl trimethyl ammonium chloride chitosan/*N*,*O*-carboxymethyl chitosan nanoparticles prepared by the emulsion-crosslinking method as a vaccine adjuvant/delivery system (219 ± 13.72 nm), using ovalbumin as a model antigen. While humoral and cellular immune responses were seen following both intramuscular or oral administration of vaccines, sIgA immunity was elicited only in orally immunized mice, confirming the ability of the developed chitosan-based adjuvant/delivery system to elevate the immune responses of the protein antigens through the oral route.

Another approach investigated to enhance the performance of chitosan-based systems for vaccination includes surface modification with other polymers such as polyethylene glycol (PEG), poloxamer, Eudragit^®^, and sodium alginate. Specifically, through polymer coating, it is possible to increase the stability of chitosan nanoparticles in physiological fluids, obtain better controlled release of antigen, and further improve mucoadhesive characteristics [206,207]. However, the physicochemical and structural characteristics of materials used for coating are of great importance. For example, in a recent study, chitosan nanoparticles, loaded with ovalbumin were coated with PEG or sodium alginate [208]. The hydrophilic polymer PEG was selected due to its ability to form a shielding layer around chitosan nanoparticles, thereby enhancing its stability in the gastrointestinal system. On the other hand, sodium alginate is a negatively charged mucoadhesive polymer. While coating with either polymer resulted in the formation of negatively charged particles, it was reported that alginate-coated chitosan nanoparticles were more stable in simulated gastric and intestinal fluid than PEG-coated chitosan nanoparticles and showed sustained release of protein. Alginate coating of chitosan-selenium nanoparticles for the delivery of lipopolysaccharides derived from *Vibrio Cholerae* [209] and chitosan nanoparticles loaded with hepatitis B antigen improved cytotoxicity and resulted in the generation of better immune responses [198].

In a study carried by Buffa et al. [210], chitosan and various TLR ligands were delivered by different administration routes (sublingual, intranasal, intravaginal, and subcutaneous) in mice, and systemic and mucosal immune responses against HIV-1 CN54gp140 and tetanus toxoid were evaluated. Sublingual immunization with chitosan significantly improved systemic immune responses to tetanus toxoid and strong Th2 biased effect for both antigens. Nasal immunization generated a more balanced Th1/Th2 profile, whereas the intravaginal route was less effective. These findings emphasize the importance of administration route, antigen type, and adjuvant system on the stimulation of immune responses [210]. Interestingly, methylglycol chitosan and a synthetic toll-like receptor 4 agonist (CRX-601) were assessed sublingual immunization with a monovalent detergent-split flu virus vaccine. It was observed that sublingual immunization with either methylchitosan or CRX-601 vaccine formulation resulted in significantly greater specific serum IgG and mucosal IgA titers compared to non-adjuvanted vaccine and were equivalent to or greater than titers obtained following i.m. vaccination [211].

Chitosan-based systems have also been investigated for oral vaccination of animals and fish in several studies [213,214,216,244].

## 6. Chitosan for Prevention and Treatment of COVID-19

Coronavirus disease (COVID-19) is an infectious disease caused by the SARS-CoV-2 virus that emerged in late 2019 and an outbreak was declared by WHO as a pandemic on 11 March 2020 [245]. COVID-19 affected the lives of millions of people all around the world, causing a public health emergency and socio-economic problems. At the beginning of pandemics, research activities focused on the repurposing of known drugs for treatment of COVID-19. In parallel with these studies and vaccine studies, rapid action was taken to develop COVID-19 vaccines as the main measure to reduce the risk of severe disease [246,247]. Currently, there are numerous antiviral drugs on the market for treatment of COVID-19 as well as vaccines for prevention [248,249].

During the pandemic period, the potential of chitosan and its derivates to combat COVID-19 both as drug delivery and vaccine adjuvant/delivery has also been considered widely (Table 5 and Table 6). Its antiviral effect made chitosan more attractive.

During the early months of the pandemic, chitosan in nanofiber form was incorporated into textiles and fabrics for prevention [250]. Due to positive charge, the nanofiber-based gowns or masks could allow electrostatic repulsion of virus particles and consequently reduce the transmission of the virus. It was proposed that the interaction of the cationic chitosan derivative, *N*-(2-hydroxypropyl)-3-trimethylammonium chitosan chloride, with a virus that has a negative charge would reduce the viral entry. The antiviral activity was shown to be influenced by the degree of deacetylation [251]. Further studies using human airway epithelium showed that the blocking of the virus would be due to interaction between the polymer and S protein of the virus [252].

As SARS-CoV-2 virus enters the body mainly through the nasal mucosa [253], effective inhibition of virus at nasal portal of entry would reduce the risk of infection. In this regard, various systems for nasal delivery have been developed based on chitosan for treatment of COVID-19 [254,255,256,257]. Chitosan was used alone or in combination with other antiviral drugs (Table 5).

**Table 5:** Summary of the studies on chitosan-based drug delivery systems for treatment of COVID-19.

Drug	Chitosan Properties	Delivery System	Aim	In Vitro Studies	In Vivo Studies	**Results**	**Ref.**
Chitosan itself	Chitosan (Mw 221,620 Da), Chitosan oligosaccharide (Mw 15,604 Da), Water-soluble chitosan (Mw 84,504 Da)	Solution	Antiviral effect against SARS-CoV-2	–Antiviral activity against SARS-CoV-2 by quantitative viral RNA targeting the RdRp and E genes and plaque assay –Cytotoxicity in Vero cells		–Low cytotoxicity for all chitosans –Antiviral effect in virus-infected cells in a dose-dependent manner for chitooligosaccharide	[258]
Hydroxychloroquine	Chitosan oligosaccharide (COS) (Mw < 2000 Da, deacetylated)	Hydrogel	Drug delivery		Model: Hamster Dose: –25 mmol/L COS solution –COS (0.4% *w*/*w*) + HCQ (0.65 mg/kg) mixture –HCQ (1.3 mg/kg) –Viral load –Pathology	–COS + HCQ group showed the lowest viral loads and highest IL-10, IgA, and IgG levels on day 8, and the highest IL-6 levels on day 4 –Earlier inflammation resolution and swifter viral clearance –Increased HCQ absorption –No evidence of cardiac or hepatic injury	[259]
Favipiravir	Chitosan (Mw 73 kDa; DD: 91.7%)	Chitosan-alginate nanoparticles (233 nm)	Nasal drug delivery	–Mucoadhesion –Drug release –Transmucosal permeation –Viral infectivity in PEDV as a surrogate virus for SARS-CoV-2		–Increased interaction with mucus of chitosan-coated NPs compared to uncoated –Higher permeation and deposition in nasal mucosa –Inhibition of viral replication	[260]
Chemically synthesized antiviral peptides (Pep1 and Pep2), each consisting of three amino acid residues	Chitosan hydrochloride (Mw: 42.7 kDa; DD: 88%)	Chitosan-hyaluronic acid nanocomplexes: Small-sized NPs (Pep1-NPs: 186 ± 2.43 nm; Pep2-NPs: 184 ± 3.12 nm) Large-sized NPs: (Pep1-NPs: 380 ± 5.1 nm; Pep2-NPs: 420 ± 6.2 nm)	Nasal drug delivery	–Cytotoxicity in Vero cells –Antiviral activity		–Lowest IC_50_ values against SARS-CoV-2 for small-sized NP (200 nm)-based delivery compared to larger NPs (300–400 nm) –Neutralization of virus –Biocompatible	[261]
Silymarin	Low Mw chitosan	Silymarin–chitosan nanoparticles (82 nm)	Drug delivery	–Antiviral activity in Vero E6 cells –Cytotoxicity in Vero and Vero E6 cell lines –Drug release		–Improved antiviral activity –Lower cytotoxicity –Initial burst release followed by slow release (87% drug released in 24 h)	[262]
Curcumin	Chitosan	Chitosan-coated-D-alpha-tocopheryl polyethylene glycol succinate bilosomes (256 ± 18 nm)	Drug delivery	–Cytotoxicity and cellular uptake in Caco-2 cells –Antiviral activity in VERO-E6 cells –Molecular docking studies –Drug release		–Biocompatible –Improved cellular uptake –8.3-fold higher anti-SARS-CoV-2 activity –Direct inactivation on surface spike protein (hACE2) –Interaction of curcumin with key residues of the virus enzymes –Increased release rate with chitosan nanocarrier	[263]

COS: chitosan oligosaccharide; DD: deacetylation degree; HCQ: hydroxychloroquine; IC: inhibitory concentration; kDa: kilodalton; Mw: molecular weight; nm: nanometer; PEDV: porcine epidemic diarrhea virus; SARS-CoV-2: severe acute respiratory syndrome-related coronavirus-2.

Pyrć et al. [254] investigated a low molecular weight chitosan derivative, *N*-palmitoyl-*N*-monomethyl-*N*,*N*-dimethyl-*N*,*N*,*N*-trimethyl-6-*O*-glycol chitosan (GCPQ), as a prophylactic nasal spray for SARS-CoV-2 infection. In the transgenic mouse model, the nasal application of GCPQ resulted in trends towards the inhibition of viral replication in the respiratory tract and brain. Moreover, due to its mucoadhesive property, it was reported that prolonged residence time in the nares was provided, which is an advantage for use in viral inhibition.

Chitosan was also used in combination with other polymers. In a study by Hanafy et al. [264], albumin nanoparticles loaded with plant extracts were coated with chitosan, and the developed mucoadhesive system was delivered by inhalation. The antiviral activity against SARS-CoV2 was demonstrated in vitro, and the reduction in inflammation was demonstrated in vivo in mice with triggered lung injury. In another study where the immunogenicity of a receptor-binding domain (RBD) of SARS-CoV-2 spike glycoprotein loaded into N,N,N-trimethyl chitosan nanoparticles (RBD-TMC NPs) was investigated, robust systemic responses and significantly higher local responses were obtained in mice following nasal administration [257].

On the other hand, taking into consideration the successful results obtained previously with chitosan, it has been investigated as a vaccine delivery system and adjuvant against COVID-19 [56,265]. Although there are approved vaccines against COVID-19 on the market, there is still a need for vaccines that prevent transmission and provide both long-term and broad-spectrum protection against the virus variants [266]. Moreover, safety is still a crucial requirement.

In Table 6, the studies on the development of vaccines based on chitosan against COVID-19 are summarized. In general, the spike protein of SARS-CoV-2 or its receptor-binding domain (RBD) produced in Pichia pastoris have been the most commonly used antigens in these systems. Chitosan was used alone or in combination with other polymers or adjuvants.

**Table 6:** Summary of the studies on chitosan-based vaccine formulations against COVID-19.

Antigen	Chitosan Type	Delivery System	Additional Adjuvant	Delivery Route	In Vitro Studies	In Vivo Studies	Results	**Ref**
S1 subunit of spike protein of SARS-CoV-2 virus OVA (as model antigen)	Fluorocarbon modified chitosan (FCS) synthesized using chitosan (DD: ≥95%)	Nanocomplexes: –FCS/S1/polyIC (2:1:1) nanocomplexes: (200 nm) –FCS/OVA nanocomplexes: (195 nm)	polyIC	transdermal		Immunization in mice Dose: 20 μg Boosters: Days 7, 14, and 42 –S1-specific antibody titers –T cells in spleen, skin, and LNs –Antigen accumulation in LNs, maturation of DC, and activation of CD8+ T cells	–Similar antibody titers to that of sc with transdermal delivery for 42 days –Increased IFN-γ levels with transdermal delivery –Long-term adaptive immune memory effect triggered	[267]
Receptor-binding domain (RBD) protein of SARS-CoV-2	*N*,*N*,*N*-trimethyl chitosan	*N*,*N*,*N*-trimethyl chitosan NPs: (386.5 ± 58.9 nm)			–Cytotoxicity assay and cellular uptake study in primary human nasal epithelial cells (HNEpCs) –Cytokine and chemokine production –Expression of maturation markers on monocyte-derived dendritic cells (MoDCs)		–No toxicity up to 100 µg/mL –Facilitated delivery of antigens into HNEpCs –Innate and adaptive immune responses obtained –Increased production of pro-inflammatory cytokines (IL-6, TNF-α, and IL-1β), chemokines (IL-8 and MIP-1β), Th-1-related cytokines (IFN-γ and IL-12), and growth factors (G-CSF and GM-CSF). –Upregulation of CD80, CD83, CD86, and HLA-DR expression in MoDCs	[268]
Vesicular stomatitis virus (VSV)-based constructs bearing spike proteins from different SARS-CoV-2 strains	Chitosan	Recombinant viral vector vaccine with chitosan		oral		Immunization in female golden hamsters (6–8 weeks old) Dose: 10^6^ TCID_50_ Booster: Day 14 –IgA and IgG levels –Cytokine and chemokine mRNA Challenge test with SARS-CoV-2 WT, Beta, Delta, and Omicron variants BA.1 and BA.2 (1 × 10^3^ TCID_50_) (intranasal)	–Both local and systemic antibody responses stimulated –Increased cytokine and interferon-stimulating gene levels –Long-lasting neutralizing antibodies induced for one year –Reduced SARSCoV-2 replication in the upper and lower respiratory tracts and alleviated lung lesions –Increased SARS-CoV-2-specific sIgA in the digestive tract	[269]
Plasmid DNA-encoding spike (S) or nucleocapsid (N) protein	Chitosan oligosaccharide	Mixture of chitosan oligosaccharide pDNA encoding S and chitosan oligosaccharide pDNA encoding N (159.87 ± 1.52 nm)		i.d. and i.m.	Cytotoxicity in HEK-293T cells, DC 2.4 cells, and Raw 264.7 cells –Uptake and transfection efficiency in HEK-293T cells, –Evaluation of DCs activation and antigen presentation	Microneedle-mediated immunization in female BALB/c mice (6−8 weeks old) Dose: 20 μg (10 μg for each plasmid) Booster: Weeks 2 and 4 –SARS-CoV-2 spike-specific antibody titers –Neutralizing antibodies –IFN-γ levels	–Neutralizing antibody levels comparable to those of i.m. immunization –Enhanced systemic and mucosal immune responses	[270]
Plasmid encoding the S protein of SARS-CoV-2	Chitosan	Gold-nanostarchitosan nanocarrier (AuNS-CS-NPs) (35–48 nm)		i.n.	Quantitative determination of cellular uptake in A549 cells	Immunization in BALB/c and C57BL/6J mice Dose: 20 μg Booster:Day 14 –Serum neutralization assay with pseudotyped lentivirus –SPK-specific IgG, IgM, and IgA antibodies in blood –Histology of lungs, spleen, and LNs –Immunophenotyping	–High and consistent antibody levels for several weeks –Effective neutralization intranasal delivery –Activation of humoral pulmonary immune responses –Increased anti-SC2 IgA levels in lung mucosa –Long-lasting immunity	[256]
Spike protein receptor-binding domain (RBD)	Mannose-conjugated chitosan	Mannose-conjugated chitosan NPs (290 ± 18 nm)	CpG55.2	i.n.		Immunization in 4-to-6-week-old female BALB/c mice Dose: 5 µg Booster: Day 21 –Humoral and cellular immune responses Immunization in 6-to-8-week-old male Syrian hamsters Dose: 5 µg Booster: Day 21 Challenge: with SARS-CoV-2 (1× 104 TCID50) intranasally –Virus transmission –Viral titers and histological evaluations –Alum-adjuvanted RBD protein administered i.m. for comparison	–sIgA and Th1-cell-mediated responses obtained only with i.n. delivery but not with i.m. –Effective protection and reduced viral load in the nasal turbinates and oropharyngeal, but virus transmission was not prevented.	[255]
S proteins derived from SARS-CoV-2 wild-type (Wuhan-Hu-1) and Delta (B.1.617.2) variants	*N*-(2-Hydroxy)-propyl-3-trimethyl ammonium chitosan chloride (HTCC) with 90% substitution	2-hydroxypropyl-trimethylammonium chloride chitosan and amylose entangle Au NPs (HTCC/amylose/AuNPs) (40 nm)		i.m.	–Cytotoxicity in Raw 264.7 and IPEC-J2 cells –Cellular uptake in RAW 264.7 cells	Immunization in BALB/c female mice (6−8 weeks old) Dose: 5 μg Booster: Day 14 –IgG and its subtypes –Neutralizing antibody levels –Cell surface markers –Biocompatability	–Improved cellular uptake –Low cytotoxicity –Increased IgG, IgG1, and IgG2a and neutralized antibody levels –Robust T- and B-cell immune responses obtained –Increased cellular responses –Biocompatible	[271]
Inhalable spike protein S	Chitosan (Mw: 190–310 kDa; DD: 75–87%)	Chitosan NPs (210.3 ± 2.1 nm)		inhalation	–Cell viability in DC2.4 –Immune activation on bone marrow derived dendritic cells (BMDCs)	Immunization in BALB/c female mice (6−8 weeks old) Dose: not stated Booster: Weeks 2 and 4 –Immune responses in lungs and bronchoalveolar lavage fluid –Histopathological analysis	–Increased IgG levels –Mixed Th1/Th2 immune responses obtained indicating predomination of Th2 immunity –High IgA levels in BALF –Increased Spike-specific T cell responses	[272]
Spike glycoprotein of SARS-CoV-2 produced in *P. pastoris*	*N*,*N*,*N*-trimethyl chitosan (Mw not stated)	*N*,*N*,*N*-trimethyl chitosan NPs (343.3 ± 3.4 nm)		i.p.	–Uptake of NPs by THP-1 cells	Immunization in female BALB/c mice aged 6–8 weeks old Dose: 10 or 20 µg Boost: on days 15 and 30 –Detection of antigen-specific antibodies (IgG, IgG1, IgG2a, IgA) –Assessment of levels of neutralizing antibodies against SARS-CoV-2 –Assessment of cellular immune responses	–Higher uptake with nanoparticles –Increased IgG and IgA levels –Th-1 and Th-2 immune responses –Higher neutralizing antibody levels –Induction of systemic cell-mediated immunity	[273]
Receptor-binding domain (RBD) of SARS-CoV-2 spike glycoprotein produced in *P. pastoris*	*N*,*N*,*N*-trimethyl chitosan (Mw not stated)	*N*,*N*,*N*-trimethyl chitosan NPs (386.5 ± 58.96 nm)		i.n.		Immunization in female BALB/c mice (6–8 weeks old) Dose: 10 or 20 µg Booster: Days 8, 15, and 30 –RBD-specific antibodies in sera, lung homogenates, and BA –In vitro virus neutralization	–Higher IgA levels in lung –Highest IgG and IgA levels in BAL at 20 μg antigen dose –Higher systemic humoral responses at 20 μg antigen dose –Th-1 and Th-2 responses obtained –Dose-dependent cell-mediated immune responses induced	[257]
Receptor binding domain (RBD) of spike (S) glycoprotein	Chitosan	Zn-chitosan-adjuvanted formulation Compared to Alhydrogel- and Adju-Phos-adjuvanted formulations		i.p.		Immunization in 5–6 week old mice Dose: 30 µg followed by 20 µg of booster dose Booster: Weeks 2 and 5 –Antibody responses –Virus neutralizing activity –Antibody avidity	–Similar antibody responses in all groups after 1st immunization –Higher immune responses after booster –Neutralization titers after first dose –Protection for 5 months	[274]
pDNA encoding spike (S) and nucleocapsid (N) of SARS-CoV-2		Quil-A-loaded chitosan (QAC) NPs loaded with pDNA (pQAC-CoV)	Quil A	i.n. and i.m.		Immunization in C57BL/6 mice (6 weeks old) *Homologous vaccine strategy with pQAC-CoV* Dose: 75 µg/ plasmid DNA/mouse Booster: Weeks 3 and 6 *Heterologous vaccine strategy (pQAC/MVA-CoV)* Dose: 75 µg/ plasmid DNA/mouse Booster: Week 6 with Modified *Vaccinia Ankara* expressing S and N antigens of SARS-CoV-2 (MVA-CoV) –IgG and IgA levels –Neutralizing antibody titers in sera and BAL –Cellular immune responses	–Increased S-specific and S receptor-binding domain (RBD)-specific IgG levels and S-specific IgA levels with heterologous vaccines (pQAC/MVA-CoV) –Local and systemic neutralizing antibody induction with heterologous vaccine –Comparable or higher neutralization titers –Significant type 1 and type 17 cellular responses	[275]
Receptor binding domain (RBD) of spike protein	Chitosan (Low Mw, 25–190 kD)	Chitosan + RBD nanocarriers (227 ± 46.9 nm) Other nanocarriers: PEI + RBD (15.6 ± 0.38 nm), DOTAP+RBD (72.0 ± 4.3 nm), Neutral liposomes + RBD (155.3 ± 0.95 nm) Anionic liposomes + RBD (126.1 ± 0.2 nm)		i.n. or i.m.	–Antigen uptake in DCs using dextran as mock antigen	Immunization in NIH mice Dose: 5 µg Boost: On days 7 and 21 –RBD-specific serum antibodies –Neutralization activity –Memory T cells in the lymph nodes –Pathological evaluation	Increased IgM, IgG, IgG1, IgG2a, and IgG2b responses and higher neutralization after i.n. or i.m. vaccination with chitosan-based nanocarriers compared to anionic and neutral liposomes –Increase uptake in the presence of chitosan –Increased cellular immune responses –Biocompatible	[276]
Spike protein epitope of SARS-COV-2 displayed in Cowpea mosaic virus	Chitosan as low Mw (250 kDa), medium Mw (1250 kDa), and high Mw (1500 kDa)	Chitosan glycerophosphate hydrogels		s.c. injection	–Gelation time –Hydrogel swelling and degradation –In vitro release of CPMV	BALB/c female mice (7−8 weeks old) Dose: 100 μg Booster: Week 2 –Antibody (IgG1, IgG2a, IgG2b, IgG, IgG3, IgA, IgE, IgM) levels	–Better in vitro results with HMW –Slow in vitro and in vivo release of CPMV –Sustained antibody response over 20 weeks –Th2-biased response	[277]

CPMV: Cowpea mosaic virus; DCs: dendritic cells; DD: deacetylation degree; FCS: fluorocarbon modified chitosan; i.d.: intradermal; i.m.: intramuscular; i.n.: intranasal; i.p.: intraperitoneal; kDa: kilodalton; LNs: lymph nodes; MoDCs: monocyte-derived dendritic cells; Mw: molecular weight; nm: nanometer; NP: nanoparticle; OVA: ovalbumin; *P. pastoris: Pichia pastoris*; RBD: receptor binding domain; s.c.: subcutaneous; SARS-CoV-2: severe acute respiratory syndrome-related coronavirus-2.

Nkanga et al. [277] have developed injectable hydrogels using chitosan of different molecular weights to deliver spike protein epitopes of SARS-CoV-2 displayed in the Cowpea mosaic virus, which provide slow antigen release and prolonged immunostimulation. Prolonged release was obtained with high molecular weight chitosan. Antibody responses were obtained over 20 weeks after s.c. administration.

In another study, it was shown that chitosan nanoparticles (227 ± 46.9 nm) loaded with RBD were superior to anionic and neutral liposomes in stimulating increased IgM, IgG, IgG1, IgG2a, and IgG2b responses following intranasal and intramuscular vaccination [276].

Chitosan derivatives have also been investigated against COVID-19. The trimethylated derivative in nanoparticle form loaded with spike glycoprotein of SARS-CoV-2 was investigated in mice following subcutaneous administration [273]. The uptake of the antigen as well as the systemic and mucosal immune responses were shown to be significantly increased in the presence of chitosan. Later, the same group showed that these nanoparticles induced mucosal and systemic immune responses against SARS-CoV-2 following nasal immunization as well [257].

## 7. Perspectives for the Future

In recent years, chitosan and its derivatives have been recognized as a potential material for delivery of a wide range of therapeutic agents and antigens due to their promising properties, such as bioadhesivity and penetration-enhancing effect, as well as their cationic structure, which makes them different from other polymers investigated. Furthermore, with its bioactive properties, specifically anti-inflammatory, antimicrobial, antiviral, tissue regeneration, and immunostimulatory activities, chitosan can be used alone or in combination with other drugs in the treatment of infectious diseases. Studies have demonstrated that chitosan and derivatives have also potential in the prevention of infectious diseases, both as an adjuvant and antigen delivery system. Chitosan has been very attractive, especially for non-invasive vaccine delivery. However, despite such attractive properties and an enormous number of studies conducted, there are currently no drug products containing chitosan that have reached the market yet. The only commercially available chitosan-related products are non-woven or woven wound dressings in the form of gels, sponges, granules, fibers, or gauze. These products are registered as medical devices. A standard guide for characterization and testing of chitosan salts as starting materials intended for use in biomedical and tissue-engineered medical product applications has been released by the FDA, addressing key parameters relevant for the functionality, characterization, and purity of chitosan salts. In Europe, according to the classification of medical devices in use by the EU medical device legislation (MDR), which is a risk-based system, products based on chitosan are considered as Class III devices; therefore, manufacturers have to provide data of clinical evaluation and/or investigation.

Chitosan for use in foods is considered under “GRAS” (generally recognized as safe) status in the US market. Yet, for chitosan-based drug delivery systems, from a regulatory point of view, meeting the quality, safety, and efficacy requirements still remains a challenge, due to their standardization, and there are still concerns over the source, purity, and immunogenicity of chitosan. Although, there are compendial monographs for chitosan in the USA, European Union, and Japan, it is still not easy for the manufacturing companies to provide the same quality with every batch, and depending on the source obtained, the properties show variability, which also effects the safety and quality of chitosan. Purification of chitosan is very important, especially due to the potential for allergenicity based on the presence of shellfish protein in sensitized individuals. Furthermore, being a naturally derived polymer, chitosan is susceptible to endotoxin contamination, which may cause pro-inflammatory responses resulting in undesired effects. Hence, it is critical that endotoxins are quantified and removed for in vivo use. With this context, there is still a need for more clinical studies showing the safety of chitosan and derivatives as biomaterials for drug delivery.

Nevertheless, due to its inherit properties as well as the promising results obtained for drug and vaccine delivery, with awareness of challenges related to purification, standardization, and manufacturing, efforts are being continued intensively in collaboration with the regulatory bodies. Especially as an adjuvant and delivery system for vaccines, chitosan has great potential.

In this regard, we believe that translation of chitosan-based products for prevention and treatment of infectious diseases from bench to market will not be too far from today, provided that more clinical studies are completed, especially demonstrating its safety.

## Figures and Tables

**Table 1:** Clinical trials using chitosan as a biomaterial for its antimicrobial, wound healing, anti-inflammatory, hemostatic, and immunostimulatory activity (listed in ClinicalTrials.gov).

Trial Title ClinicalTrials.gov ID	Sponsor	Study Aim	Status
Diode Laser and Photodynamic Therapy Vs. Ciclopirox NCT05809297	Universidad Complutense de Madrid (Madrid, Spain)	To compare the efficacy of treatment of onychomycosis by diode laser combined with photodynamic therapy and topical treatment with Ciclopirox Hydroxypropyl Chitosan (HPCH) Nail Lacquer.	Not yet recruiting
Photodynamic Therapy and Topical Antifungal for Onychomycosis in Patients With Diabetes NCT06485050	Universidad Complutense de Madrid (Madrid, Spain)	To augment topical treatments in routine clinical practice with adjunctive therapies such as photodynamic therapy for the treatment of onychomycosis in patients with diabetes.	Active, not recruiting
Antimicrobial Efficacy of Nano-Based ICM on E.F NCT06533215	Tanta University (Tanta, Egypt)	To evaluate the antimicrobial effect of nano-based intracanal medications (triple antibiotic-loaded chitosan nanoparticles and chlorohexidine-loaded silver nanoparticles) on *Enterococcus faecalis* count reduction in secondary endodontic infection cases.	Recruiting
PLGA Nanoparticles Entrapping Ciprofloxacin to Treat *E-Fecalis* Infections in Endodontics NCT05475444	British University in Egypt (Cairo, Egypt)	To treat root canals of patients suffering from bacterial infection by PLGA nanoparticles coated with chitosan polymer incorporated into in-situ gel.	Completed
Clinical and Radiographic Evaluation of LSTR in Non-vital Primary Molars Using Two Different Vehicles (LSTR) NCT05079802	Ain Shams University (Cairo, Egypt)	To compare the clinical and radiographic success of LSTR (Lesion Sterilization and Tissue Repair) in the study group where a double antibiotic mix is mixed with chitosan nanoparticles and the control group where a double antibiotic paste is mixed with propylene glycol.	Not known
Efficacy and Safety of the Investigational Device, SurgiShield Anti-Adhesion Barrier Gel NCT01895933	D.med (ChoongChungBukDo, Republic of Korea)	To evaluate the impact, efficacy, and safety of chitosan-formulated adhesion inhibitor SurgiShield when used in the process of wound healing after endoscopic sinus surgery.	Completed
Efficacy of SynEx Wound Rinse in Civilian Surrogates of Combat Injury Wounds NCT05743283	Synedgen, Inc. (Claremont, CA, USA)	To compare SynEx Wound Cleanser (an osmotically balanced wound cleanser containing patented chitosan derivatives) with the current routine care (saline) in traumatic wounds in participants with gunshot, penetrating, or burn wounds.	Recruiting
EGF-loaded Chitosan to Facilitate Healing and Prevent Scar Formation of Cesarean Wound NCT04211597	Chang Gung Memorial Hospital (Taipei, Taiwan)	To investigate the effect of microencapsulated recombinant human EGF (Me-EGF) (brand NewEpi^®^). The investigational dressing NewEpi^®^ was formulated in spray solution with a proprietary drug delivery system in which rhEGF was encapsulated in chitosan as nanoparticles.	Completed
Slow-release Tb4 Collagen and Chitosan Porous Sponge Scaffolds Skin Substitute Treatment is Difficult to Heal Wounds (TB4) NCT02668055	Chinese PLA General Hospital (Beijing, China)	To evaluate the effectiveness and safety of slow-release Tb4 collagen and chitosan porous sponge scaffold skin substitutes for the treatment of difficult-to-heal wounds.	Completed
Chitosan Scaffold for Sellar Floor Repair in Endoscopic Endonasal Transsphenoidal Surgery NCT03280849	University of Guadalajara (Guadalajara, Mexico)	To repair the sellar lesion in a 65-year-old female participant, that compressed the optic chiasm. An endoscopic endonasal transsphenoidal surgery was performed for the resection of the lesion, using a novel bilaminar chitosan scaffold to assist the closure of the sellar floor.	Completed
Effectiveness and Safety of Early-Stage Amputation and External Herbs Chitosan for Diabetic Foot Ulcer NCT02413086	Heilongjiang University of Chinese Medicine (Harbin, China)	To promote granulation tissue regeneration and control of local infection in difficult wound healing at early-stage amputation by external therapy of herbs in the presence of chitosan.	Unknowm status
Phase 1 Norwalk Vaccine Study NCT00806962	LigoCyte Pharmaceuticals, Inc. (Bozeman, MT, USA)	Randomized Double-Blind Placebo-Controlled Phase 1, Safety and Immunogenicity Study of Two Dosages of Intranasal Norwalk Virus-like Particle Vaccine (Norwalk VLP Antigen, MPL^®^, Chitosan, Mannitol, and Sucrose) Compared to Adjuvant/Excipients (MPL^®^, Chitosan, Mannitol, and Sucrose) and to Placebo (Empty Device).	Completed
Compare the Hemostatic Effectiveness of Chitosan Gauze With Traditional Gauze on Open Wound on 10 Participants NCT03907111	Tri-Service General Hospital (Taipei, Taiwan)	To compare the effects of hemostasis gauze made by chitosan with traditional cotton gauze in bleeding time, bleeding volume, wound infection, and wound healing speed when it is used in open wound treatment.	Completed
Trial of a Novel Chitosan Hemostatic Sealant in the Management of Complicated Epistaxis NCT00863356	HemCon Medical Technologies, Inc. (Portland, OR, USA)	To investigate the efficacy of a chitosan-coated nasal packing (ChitoFlex^®^ used in conjunction with the HemCon Nasal Plug) in the management of difficult spontaneous epistaxis and to evaluate its healing effect on nasal mucosa.	Completed
Develop the First Aid Medical Supplies for Hemostasis and Bacteriostasis With Clinical Trial NCT04884919	Tri-Service General Hospital (Taipei, Taiwan)	To develop new biofiber chitosan-based hemostatic dressings for hemostasis and open wound care in the subjects undergoing a cesarean section.	Unknown status
Comparison of Chitosan, Ankaferd, and Tranexamic Acid in Dental Extraction in Liver Pre-Transplant Children NCT06457360	British University in Egypt (Cairo, Egypt)	To assess the hemostatic effect of chitosan-based dressing, Ankaferd, and Tranexamic acid after extraction in children with end-stage liver diseases.	Completed
Dental Extractions in Patients Under Dual Antiplatelet Therapy (DUALex) NCT02918045	University of Sao Paulo General Hospital (São Paulo, Brasil)	To compare two hemostatic agents (a novel chitosan-based hemostatic agent and oxidized cellulose gauze) in patients under dual antiplatelet therapy using the intraoral bleeding time after dental extractions.	Completed

## Data Availability

Not applicable.
